# Mapping the Structural and Dynamical Features of Multiple p53 DNA Binding Domains: Insights into Loop 1 Intrinsic Dynamics 

**DOI:** 10.1371/journal.pone.0080221

**Published:** 2013-11-12

**Authors:** Suryani Lukman, David P. Lane, Chandra S. Verma

**Affiliations:** 1 Bioinformatics Institute, Agency for Science, Technology and Research, Singapore, Republic of Singapore; 2 p53 laboratory, Agency for Science, Technology and Research, Singapore, Republic of Singapore; 3 Department of Biological Sciences, National University of Singapore, Singapore, Republic of Singapore; 4 School of Biological Sciences, Nanyang Technological University, Singapore, Republic of Singapore; University of Leeds, United Kingdom

## Abstract

The transcription factor p53 regulates cellular integrity in response to stress. p53 is mutated in more than half of cancerous cells, with a majority of the mutations localized to the DNA binding domain (DBD). In order to map the structural and dynamical features of the DBD, we carried out multiple copy molecular dynamics simulations (totaling 0.8 μs). Simulations show the loop 1 to be the most dynamic element among the DNA-contacting loops (loops 1-3). Loop 1 occupies two major conformational states: extended and recessed; the former but not the latter displays correlations in atomic fluctuations with those of loop 2 (~24 Å apart). Since loop 1 binds to the major groove whereas loop 2 binds to the minor groove of DNA, our results begin to provide some insight into the possible mechanism underpinning the cooperative nature of DBD binding to DNA. We propose (1) a novel mechanism underlying the dynamics of loop 1 and the possible tread-milling of p53 on DNA and (2) possible mutations on loop 1 residues to restore the transcriptional activity of an oncogenic mutation at a distant site.

## Introduction

**Figure 1:**
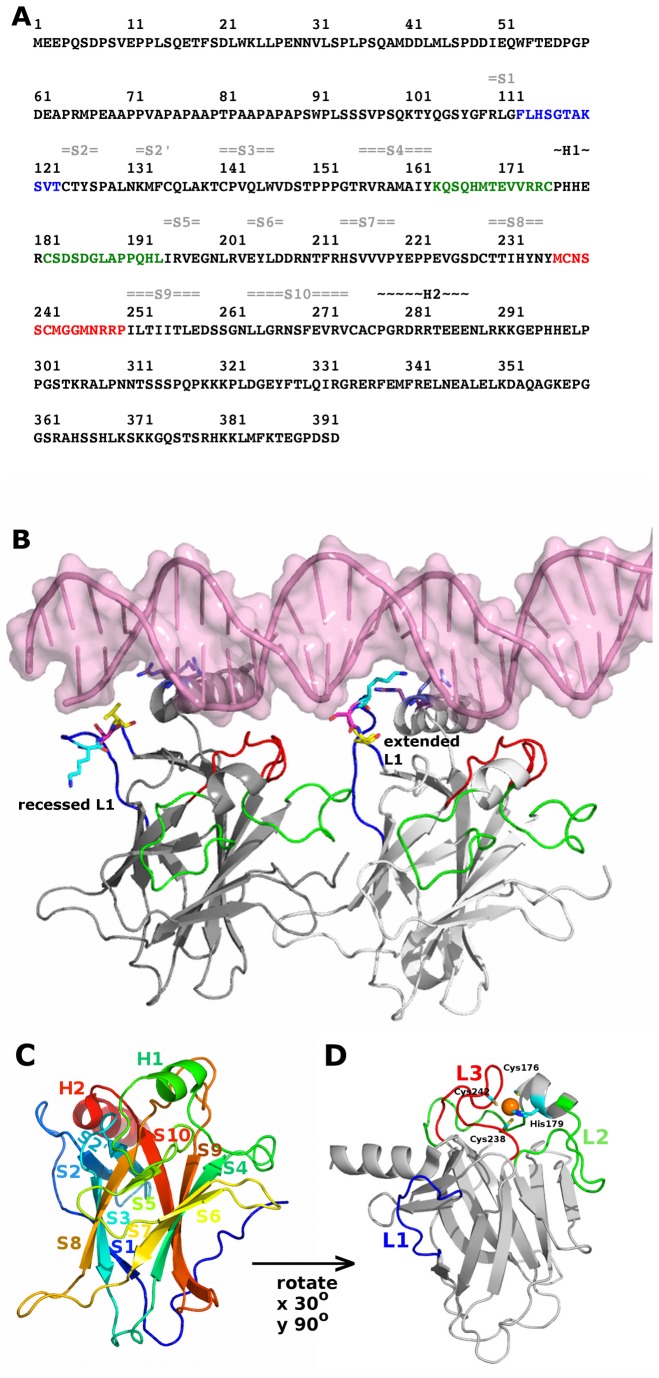
Structure of p53 DNA binding domain. (A) The primary sequence of p53 comprising 393 amino acid residues was retrieved from Uniprot P04637 isoform 1. For p53 DNA binding domain, the secondary structure contents of alpha-helices (black) and beta-sheets (gray) are indicated on top of amino acid positions. The important loops in p53 DNA binding domain: loop 1, loop 2, loop 3 are colored in blue, green, red, respectively. (B) The structures of DNA-bound p53 DBD from 3Q05 chain A (dark grey) with recessed L1 and from 3Q05 chain B (light grey) with extended L1. DNA is shown as pink transparent surface and cartoon representations. The important loops in p53 DBD: loop 1, loop 2, loop 3 are colored in blue, green, red, respectively. The following residues are shown in sticks: K120 (cyan), S121 (magenta), V122 (yellow), R280 (light blue), R283 (purple). (C) The structure of p53 DBD colored according to spectrum. Secondary structures are labeled. The PDB 2OCJ chain A was used to generate this figure. (D) The same figure as (C) except that only loop 1, loop 2, and loop 3 are colored in blue, green, and red, respectively. The zinc ion is shown as an orange sphere.

p53 is a transcription factor regulating a wide variety of genes involved in DNA repair, apoptosis, senescence [1] and metabolism [1–3] in response to stress, e.g. DNA damage, telomere erosion, and hypoxia [4]. Unfortunately, in approximately half of cancerous cells, p53 is mutated and loses its tumor suppressor function [5]. The sequence of p53 (Figure 1A) can be fragmented into an N-terminal domain (NTD), proline-rich region, DNA binding domain (DBD), and tetramerization (TET) domain [6]. The largely disordered NTD (residues M1-P67) is responsible for trans-activation. The helical TET (residues G325-A355) region is the site for oligomerization (p53 is thought to function largely as a tetramer [5]). The DBD, also known as the p53 core domain (p53C), binds to sequence-specific (target) DNA at promoter regions and initiates the transcription of genes. Different definitions of residues that form the p53 DBD exist, including residues S94-T312 [7–9], S94-K292 [5,10], S95-P295 [11], T102-K292 (UniProtKB identifier: P04637-1). For this study, we adopt the UniProtKB identifier and define residues 102-292 as the DBD. 

The p53 DBD is intrinsically unstable and unfolds at just above physiological temperature (about 42-44°C) [12], rendering it susceptible to oncogenic mutations [7]. Indeed, more than 90% of oncogenic mutations of p53 are found in the DBD [8,13], hence making it an appealing target for cancer therapies which aim to stabilize the DBD and reverse the effect of mutations. Motivated by this problem, we perform a comprehensive structural mapping of all available wild type and mutant DBD structures using principal component analysis (PCA) and a set of molecular dynamics (MD) simulations on the wild type DBD to develop a deeper understanding of its structure, dynamics and function. 

Since most existing structural and biophysical studies of p53 DBD have been performed on monomeric DBD, we analyze monomeric DBD in its wild-type and mutant forms. Although p53 activates transcription most efficiently as a tetramer [14], both monomeric and dimeric p53 exist *in vivo* [15,16]. Moreover, crystal structures of the DBD in its monomeric, dimeric and tetrameric states reveal that all of them are highly similar in their DNA-binding features [17–19]. Individual DBDs, in both monomeric and tetrameric forms, are also similar in their thermodynamic stabilities [20]. 

The DBD is an approximately 25 kDa chain consisting of an immunoglobulin-like β-sandwich (two anti-parallel β-sheets) that provides the scaffold for the DNA binding surfaces (Figure 1B). The secondary structures are indicated in Figures 1C and 1D. The DNA binding region comprises the major and minor groove binding surfaces. The major groove binding surface is formed by the loop L1 (residues F113-T123) and a short helix H2 (residues P278-E287). The minor groove binding surface is formed by two loops, L2 (residues K164-C176, C182-L194) and L3 (residues M237-P250). 

Both L2 and L3 are stabilized by a zinc ion that is tetrahedrally held by the side chains of a histidine (H179) and three cysteine residues (C176, C238, and C242) (Figure 1D). The zinc ion is necessary for the thermodynamic stability of p53 DBD [12]. The loss of this zinc ion results in increased tendency for aggregation and enhanced dynamics of surrounding loops, L2 and L3, that lead to the loss of DNA binding specificity [21,22]. In particular, the zinc ion exerts its role in maintaining the local stability of L2 and holding L3 in the proper orientation for binding to the DNA minor groove. Indeed, the zinc ion has been found to be instrumental in recovering wild type activity in mutant p53, particularly the R175H and R273H mutants [23]. 

Proteins exist as inter-converting conformational states under ambient conditions and their conformational dynamics are known to be intricately linked to their functions [24] and changes in environmental conditions [25–27], such as temperature, pressure, solvent, pH, salt concentration, and binding to ligands or macromolecules (such as the zinc ion or DNA, respectively in the case of p53 DBD). In this study, we gather the available crystallographic and NMR structures of the p53 DBD and combine these with multiple copy MD simulations to examine the conformational space that is intrinsically accessible to the wild-type p53 DBD. MD simulations have been employed by several groups to study the dynamical behavior of wild-type p53 DBD [21,28] and some of its mutants [28–33]. However, to our knowledge, there has not been a systematic comparison of MD simulations and the large ensemble of available experimental structures. It is well known that small changes in sequence (such as commonly found in p53 DBD [8], Ras [34,35], etc) can be very conservative in structural differences and yet lead to very different dynamical properties. In this study, we use MD simulations to further probe into structure-dynamics-function relationships of the p53 DBD, focusing particularly on the loops. 

## Results/Discussion

### Conformational space of crystallographic and NMR ensembles

We begin by exploring the conformational space that is spanned by the available experimental structures of DBD. As of July 2011, the RCSB Protein Data Bank (PDB) contains 57 PDB entries of monomeric and multimeric p53 DBD as well as p53 DBD in complex with DNA. From these entries, we excluded structures that have unresolved residues, particularly in the mobile loop regions. The final set consists of 141 distinct conformers (105 crystal and 36 NMR conformers). The lowest sequence identity among these conformers is 84.4%. This difference of more than 15% in sequence identity arises from species differences (human versus mouse p53 DBD), but they are highly similar in structures with Cα RMSDs of 0.32- 0.59 Å. p53 DBD from species other than human and mouse were excluded because of the higher sequence differences. We did not see any separation between human and mouse p53 DBD in the first three dominant principal components (PC) space; the details of our principal component analysis (PCA) results are presented below. The final set also includes DBD with multiple mutations such as those introduced to stabilize the DBD.

**Figure 2:**
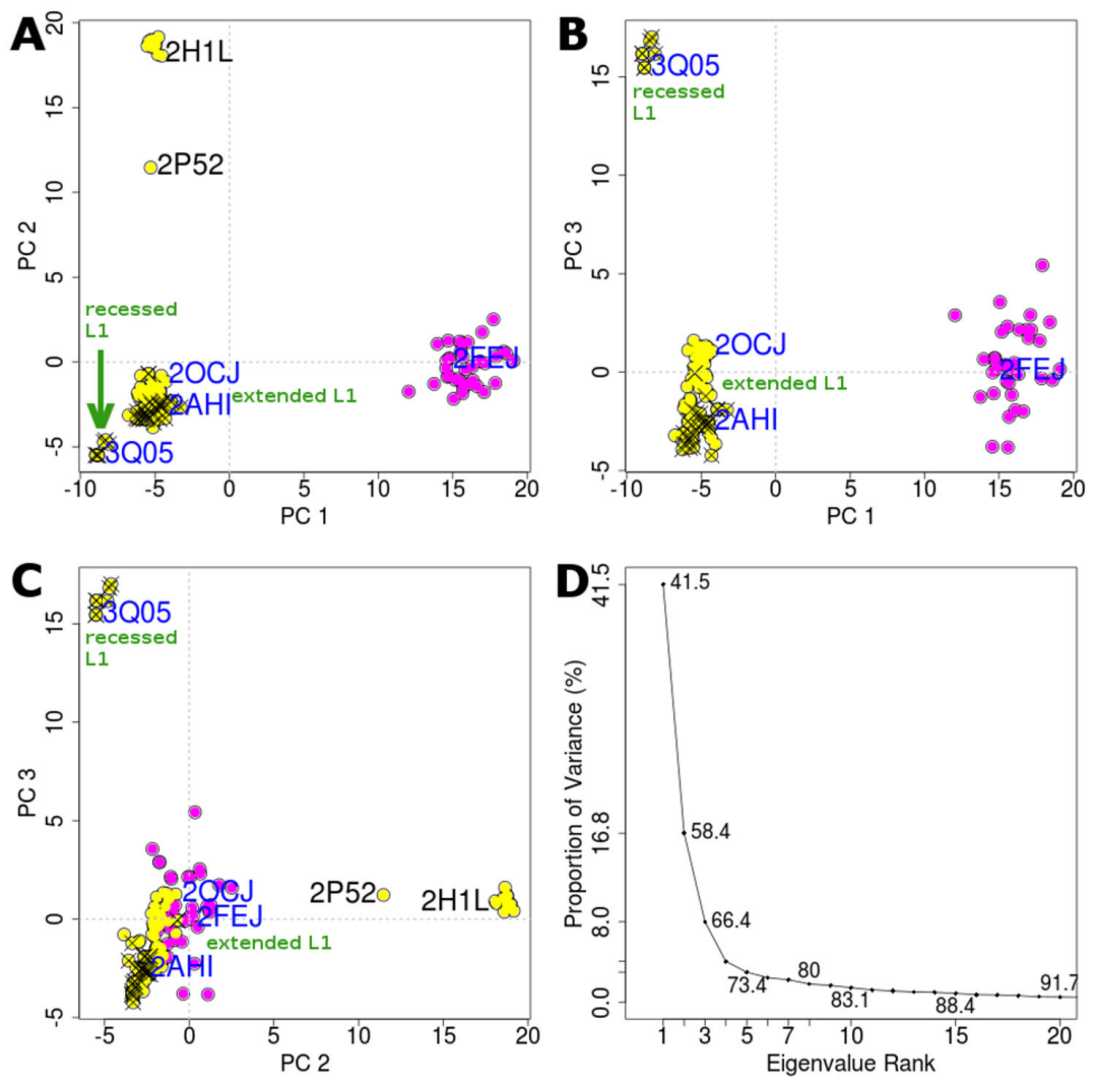
Projection of experimentally determined conformers of p53 DNA binding domain (DBD) onto the principal planes defined by the significant principal components (PC1 - PC3). (A-C) Structures determined using X-ray crystallography and nuclear magnetic resonance are colored in yellow and magenta respectively. Structures crystallized with DNA are indicated with an ‘x’. The PDB codes of experimental structures used for our subsequent molecular dynamics simulations are labeled in blue. (D) Eigenvalue spectrum obtained from the principal component analysis of the experimentally determined conformers. The magnitude of each eigenvalue is expressed as the proportion of the total variance (mean-square fluctuation) captured by the corresponding eigenvector. Labels on each point indicate the cumulative sum of variance accounted for by a particular eigenvector and its preceding eigenvectors.

To perform PCA, we first determine, through iterated rounds of structural superposition [36], 35 structurally invariant residues and these residues belong to the β-sheet secondary structures (S2', S4, S5, S7, S8, S9, S1) (Figure 1C). Both α helices (H1 and H2), β sheets S1, S2, S3, S6, and the loops are relatively flexible. PCA of the 141 conformers (Figure 2) shows that over 66% of the variance (or total mean-square displacement) of atomic positional fluctuations was captured in three dimensions, and over 58% in two dimensions (Figure 2D). Similar to observations in other systems [34,37], the first few dominant principal components (PCs) capture most of the variance in the original distribution of the p53 DBD conformational space. Dimensionality reduction to PCs enables succinct analysis of the conformational space of p53 DBD and the relationship among conformers in simple 2D planes.

The NMR and crystal conformers are well separated along PC 1. To identify residues that are responsible for the conformational differences retained by a given PC, the relative displacement of each residue along that PC is examined as atomic displacements from the mean structure (Figure S1). PC 1 describes the concerted displacement of the important loops (L1 of residues F113-T123 and L2 of residues K164-C176, C182-L194), residues prior to S1, loop of S3-S4 (residues V147-T155), loop of S6-S7 (residues D208-R213), and loop of S9-S10 (residues D259-N263). In previous structural studies using X-ray crystallography, it has been observed that L1 adopts two significantly distinct conformations when p53 DBD is bound to DNA [38,39]; these distinct L1 conformations are referred to as extended and recessed conformations. In its extended state, residues K120 and S121 of L1 make contact with DNA (Figure 1B) [17,19,38,40]; whereas in its recessed state, residue V122 of L1 (but not residues K120 and S121) makes contact with DNA (Figure 1B) [38]. For example, in a p53-DNA complex (PDB code: 3Q05), K120 of extended L1 makes double hydrogen bond contacts with Gua16 of DNA and S121 makes a hydrogen bond contact with Cyt14 of DNA, whereas residue V122 is held back from the DNA by an intra-molecular hydrogen bond with A119. In contrast, K120 of recessed L1 makes an intra-molecular hydrogen bond contact with T123, and this hydrogen bond may prevent K120 from interacting with DNA. These two states of L1 are well separated in the PC plots (Figure 2). 

Radius of gyration can be used to understand the degree of compactness of folded protein conformers. We calculated the radius of gyration, surface area and volumes of the experimentally available conformers of each DBD and plot them against PC 1 (Figure S2). PC 1 clearly separates the larger surface areas and volumes of NMR conformers from the smaller surface areas and volumes of crystallographic conformers. Given the frequent employment of cryo condition in crystallography, it is not surprising that the NMR conformers have larger surface areas and volumes than the crystal conformers, yet the PC 1 nicely shows the progression from the smallest (for crystal conformers with recessed L1), through the slightly larger (for crystal conformers with extended L1) to the largest surface areas and volumes (for NMR conformers obtained at 298K with very extended L1). The L1 clusters will be explained more in relation to PC 3. Such separation between NMR and crystal conformers has also been reported for other systems such as HIV-1 protease [37]. 

**Figure 3:**
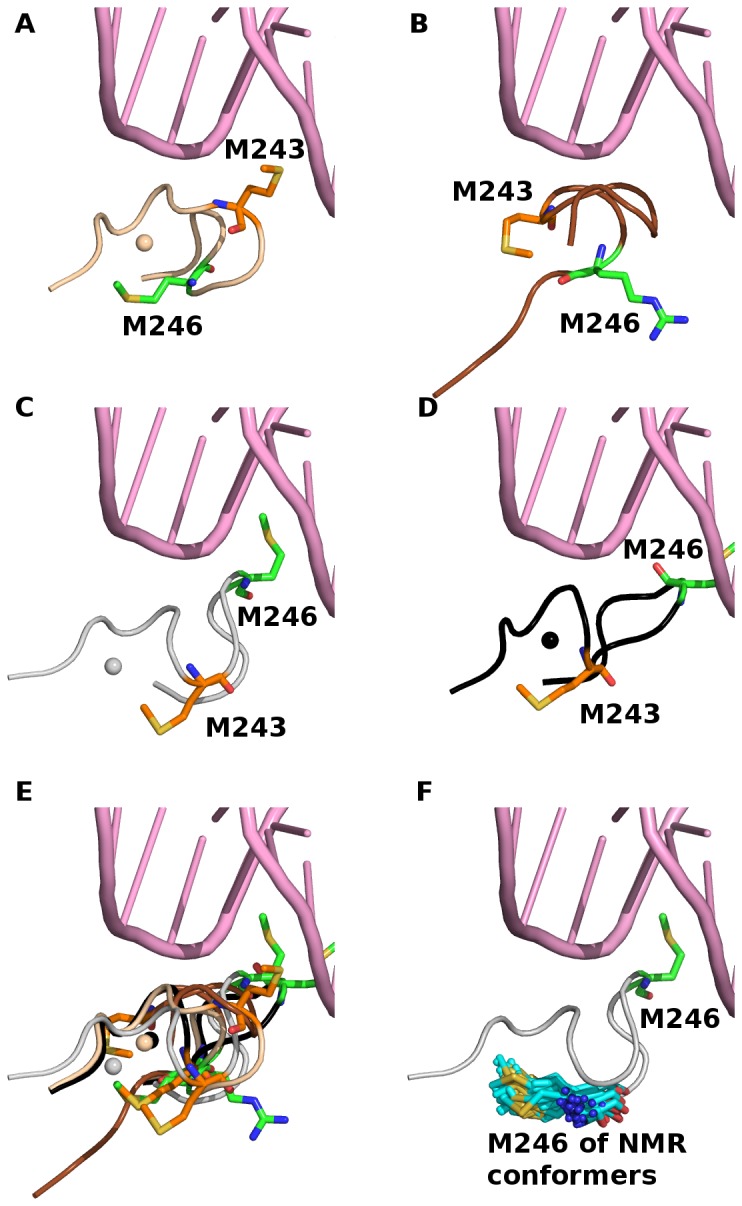
The methionine switch responsible for a large conformational change in Loop 3 is detected by PC 2. The zinc ion is shown as sphere when it is present in the respective crystal conformers (A-E). DNA (pink cartoon) is shown by aligning only the DBD of DNA-bound DBD (PDB code 2AHI) to DNA-free DBD (PDB code 2H1L and 2FEJ). (A) Loop 3 (wheat cartoon) has exposed M243 (orange stick) and buried M246 (green stick). The structure of PDB code 2AHI was used to generate this figure. (B) In the crystal structure that has no zinc ion (PDB code 2P21), the orientations of both Met residues are intermediate between (A) and (C). (C) Loop 3 (grey cartoon) has buried M243 (orange stick) and exposed M246 (green stick) in LTag-bound p53 DBD (PDB code 2H1L). For clarity, LTag is not shown. (D) In R249S mutant (PDB code 2BIO), M243 is buried and M246 is exposed on Loop 3 (black cartoon), the methionine residues are similar in conformation to the LTag-bound p53 DBD (C). (E) All structures of L3 in (A)-(D) are superimposed onto a single structure for comparisons. (F) M246 in exposed conformation from LTag-bound p53 DBD and in buried conformations from NMR resolved p53 DBD (PDB code 2FEJ) is shown in green and cyan sticks, respectively.

PC 2 separates conformers with major differences in L3 conformations; L3 is thought to be crucial for interactions of the DBD with the minor groove of DNA [5]. There are two methionine residues in the middle of L3: M243 and M246, connected by two flexible glycine residues, that appear to be instrumental in enabling conformational transitions in this region – referred to as the methionine switch (Figure 3) [41]. 

Along PC2, one of the two major clusters is made up of the crystal conformers (PDB code: 2H1L) that were crystallized with the large T-antigen (LTag) oncoprotein of simian virus 40 (SV40) [41]. The DBD co-crystallized with SV40 protein has buried M243 and exposed M246 (Figure 3C). These conformers lie in the region of PC2 > 15 (Figure 2). In contrast, members of the other cluster (PC2 < 5) have exposed M243 and buried M246. Such a switch in the two methionine residues seems to characterize the virus oncoprotein-bound DBD, in which the burial of M243 and the exposure of M246 may be associated with the inhibition of early-stage viral DNA replication [42,43]. Interestingly, the oncogenic R249S p53 DBD mutant (PDB code: 2BIO) also has buried M243 and exposed M246 in the L3 region [44] (Figure 3D). However, this structure was excluded from our PCA because it has missing residues in the L1 region. The R249S mutant has been shown to be attenuated in thermodynamic stability and has impaired DNA binding [45]. Moreover, the R249S mutation results in disrupted interactions between p53 DBD and the p53-binding protein 2 (53BP2) domain of ASPP2 [46]. The authors proposed that the replacement of Arg by Ser at position 249 removes critical interactions with the SH3 domain of 53BP2 and is largely the reason for loss of functionality, however it is possible that the methionine switch also plays a role in the disrupted interactions.

In the only NMR study reported on isolated p53 DBD, M246 seems to occupy two conformations based on the detection of line broadening [7]. This data suggests that M246 can intrinsically adopt both conformations regardless of the presence of other binding proteins such as LTag and that the conformational heterogeneity of M246 arises intrinsically. It is likely that a combination of conformational selection mechanisms and induction of conformational shift of M246 operate when LTag binds to the p53 DBD [47,48]. However, visual examination of all 36 NMR conformers did not reveal any conformation with exposed M246 akin to that of the LTag-bound p53 DBD (Figure 3F). 

Besides separating conformers with distinct methionine switch conformations in L3, PC2 also provides clues on the zinc ion. The zinc ion is coordinated by C176 of L2, H179 of H1, and C238 and C242 of L3. The crystal conformer (PDB code: 2P52), which was crystallized in the absence of zinc ion [49], lies intermediate to the two major clusters (associated with the methionine switch) along PC2 (Figure 2A,C). This result may suggest that the depletion of the zinc ion largely shifts the conformation of L3, and highlights the importance of the zinc ion in maintaining the conformation of L3 (Figure 3B). The depletion of zinc ion is associated with the oxidation of zinc-ligating cysteine residues (C176, C238 and C242). Both depletion of zinc ion and oxidation of these cysteine residues result in reduced transcriptional activity of p53 [49–51]. The presence of a zinc ion is associated with the narrowing of the minor groove of DNA upon the insertion of R248 into the minor groove, whereas in the absence of the zinc ion, no narrowing of the DNA minor groove is observed [21]. 

Similar to PC 1 in the context of L1 diversity, PC 3 captures the diversity of conformers especially at L1. Two major clusters emerge along PC 3, in which one cluster (PC 3 > 15) has recessed L1 conformations and the other has extended L1 conformations (Figure 2B,C). Only 6.67% of the crystal conformers analyzed in this study adopt recessed L1 conformations, whereas most conformers adopt extended L1 conformations. It is quite likely that the conformations of L1 will be influenced by the cryo-cooling carried out during crystallization procedure and associated effects of crystal packing [52,53]. 

### MD simulations enable enhanced sampling of the p53 DBD conformational space

To further probe the conformational space of monomeric p53 DBD, we performed two copies of MD simulations, each starting from four different structures, resulting in eight independent copies of 100 ns each. The RMSD profiles of the structural changes along the MD conformers suggest that our simulations are stable especially for the core residues (Figure S3). 

**Figure 4:**
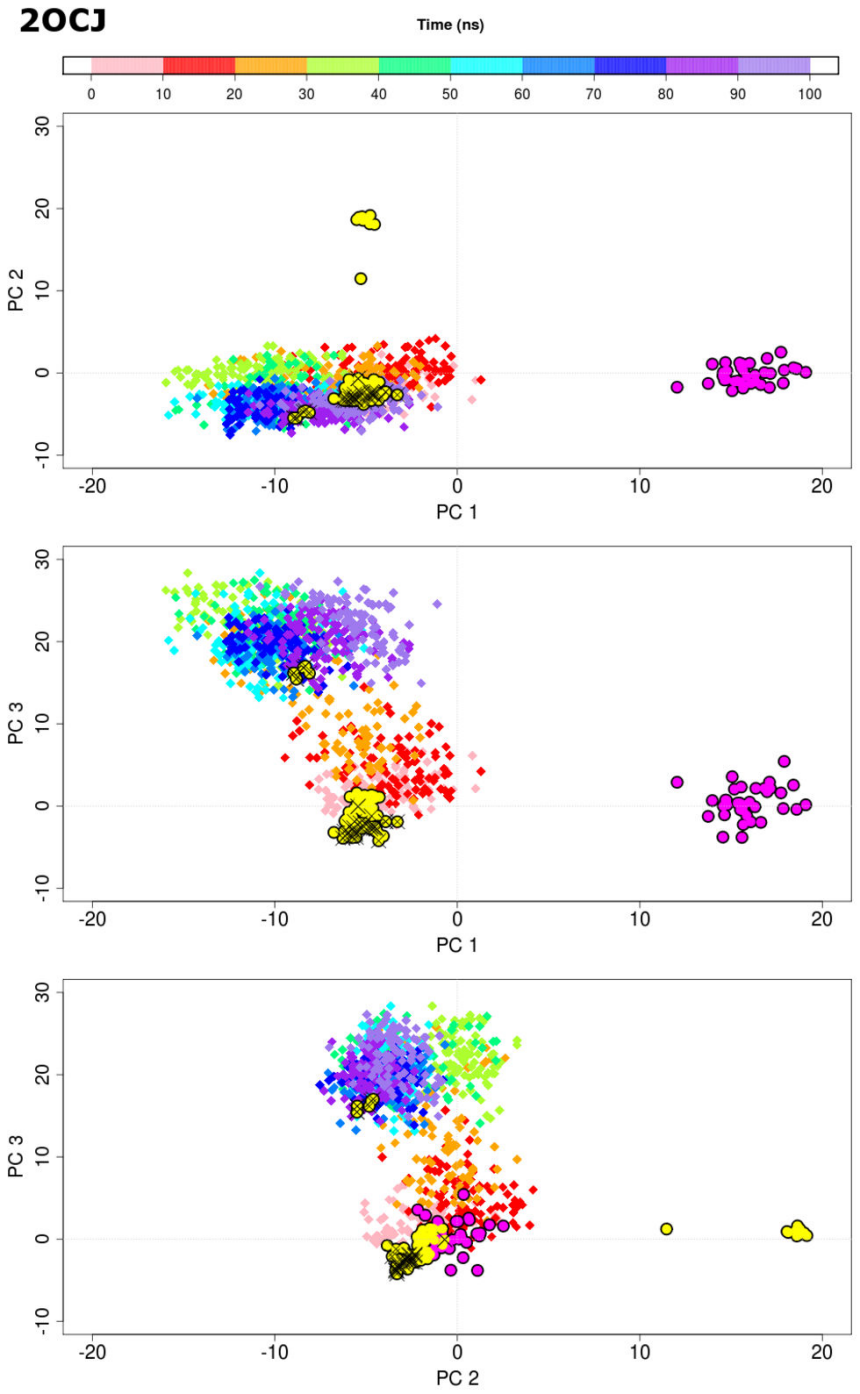
Projection of MD conformers (diamonds) onto the first three PCs defined by the experimentally-determined crystal (yellow circles) and NMR (magenta circles) structures (**see** Figure 2). The MD conformers are color mapped based on simulation time from 0 to 100 ns. The PDB code of starting structure used for each MD simulation is indicated on upper left.

We then project the MD conformers onto the principal component space defined by the crystallographic and NMR structures (Figure 4, Figures S4-S10). Having started from the wild type crystallographic structures, it is clear that at least within 100 ns (for a single trajectory), the NMR cluster is not visited by the MD, neither is the region occupied by buried M243 / exposed M246 cluster visited. However, both recessed and extended L1 conformations are accessed, regardless of the L1 states in the starting conformations. No isolated human p53 DBD has been successfully crystallized with recessed L1 conformation *hitherto*. The extended L1, particularly its K120 (in the DNA-free DBD crystal structure, PDB code: 2OCJ), seems to be stabilized by contacts formed with symmetry related molecules (Figure S11). On the other hand, we observed no contact involving K120 and symmetry related molecules in the crystal structure of DNA-bound DBD with recessed L1 (PDB code 3Q05). Though it is arguable that crystal symmetry does contribute to stabilizing the conformation of L1 in its extended state, our MD simulations (7 out of the 8 copies in Figure 4, Figures S4-S10) provide evidence that p53 DBD has intrinsic flexibility that samples both recessed and extended L1 conformational space regardless of binding to DNA. The absence of recessed L1 conformations in the DNA-free DBDs also suggests that the presence of DNA may stabilize the recessed conformations in certain conditions; simulations with DNA bound to p53 DBD are being carried out to explore this hypothesis and will be presented at a later date. 

**Figure 5:**
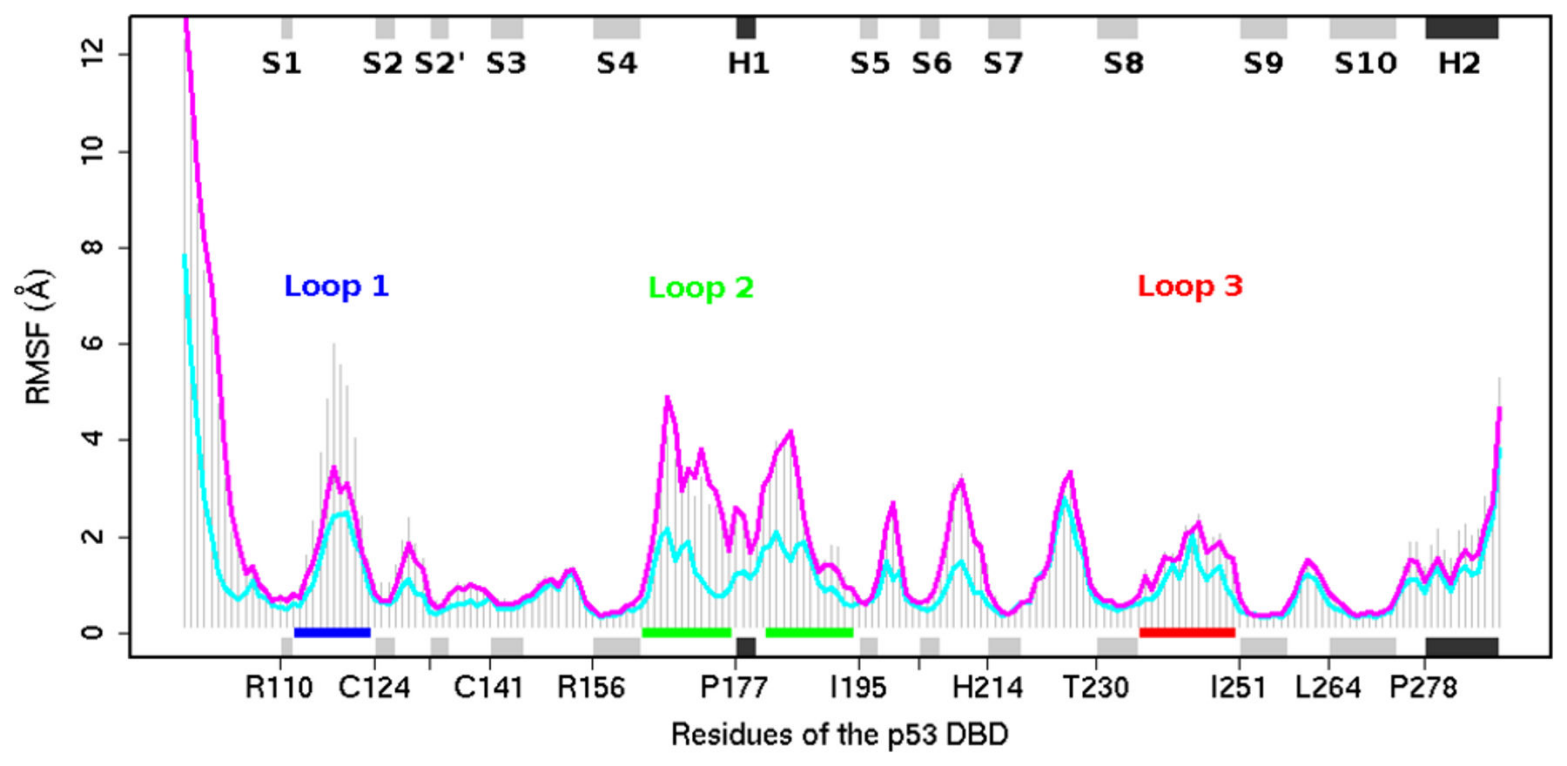
The profile of residual flexibility of p53 DBD, as indicated by root mean square fluctuation (RMSF). The RMSF for all concatenated MD conformers are shown in grey histogram. The RMSFs for extended and recessed L1 conformers are shown in magenta and cyan lines, respectively. Black and grey boxes represent alpha helices and beta sheets, respectively. L1, L2 and L3 are indicated as blue, green and red lines, respectively.

The hypothesis that DNA may stabilize the recessed L1 conformation can also be tested through energy calculations using the molecular mechanics/Generalized Born surface area (MM-GBSA) approximation. The energetic distribution of GB free energy and solvation energy of free p53 DBD conformers with extended L1 is lower than those of recessed L1 (Figure S12), i.e. in its free form, DBD with extended L1 is more stable than that with recessed L1. In contrast, we note that the molecular mechanical energy (in vacuum) of DBD with recessed L1 is more favorable (Figure S12C). This more stable molecular mechanical energy possibly accounts for the lower flexibility in loops of DBD with recessed L1 (Figure 5, discussed more in Result 4), and supports the general notion of the inverse relationship between stability and flexibility, while keeping in mind that exceptions do exist. However, the more favorable solvation energy of DBD with extended L1 compensates for its less favorable molecular mechanical energy, hence resulting in more favorable GB free energy for extended L1. This result highlights the important role of solvent (water) in the energetic distribution of p53 DBD, and hence the preferred conformations. Our calculation also provides a possible reason as to why no free p53 DBD adopting recessed L1 conformational states has been successfully obtained using experimental methods, and highlights the role of MD simulations in accessing transient or less populated conformations.

Nuclear Overhauser effect (NOE) measurements by Canadillas et al. suggested that there are compatible alternative conformations of L1 [7]. In Figure 3 of the Canadillas et al. paper, two distinct conformations of L1 are depicted, where the extended L1 is similar to L1 in DNA-bound DBD [40] and the other conformer resembles L1 in an unpublished mutant. The authors further suggested that the L1 conformational diversity spans the time scale of milliseconds to microseconds. However, among the 36 NMR conformers submitted to the PDB, we do not see any recessed L1 conformation akin to that reported in the PDB code 3Q05 (Figure S13). 

We further analyzed p53 DBD with extended and recessed loop 1 states using a simplified elastic network model that computes the normal modes of motion [54]. This technique has been widely used quite successfully in examining the links between low frequency motions and functional motions [55]. The structural mobility predicted by the NMA qualitatively suggests that a single mode (mode 3) in both cases is able to describe the transition from extended to recessed loop 1 and vice versa (Figure S17); this is in accord with observations from our MD simulations. Given that it is a low frequency mode, it is likely that this motion has a functional significance.

### Loop 1 is the most dynamic loop among L1 - L3

In order to understand the dynamical behavior of wild-type DBD as a whole, we further concatenate the eight trajectories of wild-type p53 DBD. From the RMSF profiles we see that as expected the N-terminal residues are the most flexible (Figure 5). L1 is the most dynamic loop among L1, L2 and L3.The high flexibility of L1 arises because residues H115, S116, G117 of L1 have very little interactions with the bulk protein except for a hydrogen bond between S116 and C124 of S2 [30]. Figure 5 suggests that DNA-free DBD will likely have flexible L1 and hence relatively high entropy as compared to the DNA-bound DBD. The penalty paid for sequestering L1 in the presence of DNA must be compensated for by interactions with DNA [56] i.e. the loss of the high entropy can possibly be paid for by the enthalpy acquired from the binding of p53 DBD to DNA. 

It is known that enhanced dynamics are important for molecular recognition [57]. Since L1 (together with H2) interacts with the major groove of DNA, and L1 is the most dynamic loop amongst L1-L3, we infer that L1 possibly plays an early and important role in the binding of DBD to DNA. It is possible that the major groove of DNA is targeted first prior to subsequent binding to the minor groove of DNA. On the other hand, the binding to the minor groove involves the less flexible L2 and L3, which may play secondary roles as compared to L1. This current study is unable to make a final conclusion about this hypothesis. Current efforts that utilize different methods to examine the process of DBD binding to the DNA in detail will shed more light in the future [58–61]. For example, factors such as DNA bending and post-translational modifications (e.g. acetylation) are likely to play significant roles in the process of DBD-DNA recognition. In response to DNA damage, the acetylation of K120 on L1 promotes specific binding to target DNA [62], especially to promoters of pro-apoptotic genes such as BAX and PUMA [63]. Weakly yet intuitively supporting our hypothesis, recent experimental work seems to suggest that transcription factors can distinguish their target from non-target DNA through longer, more stable binding versus tread-milling [64,65] (see below). 

**Figure 6:**
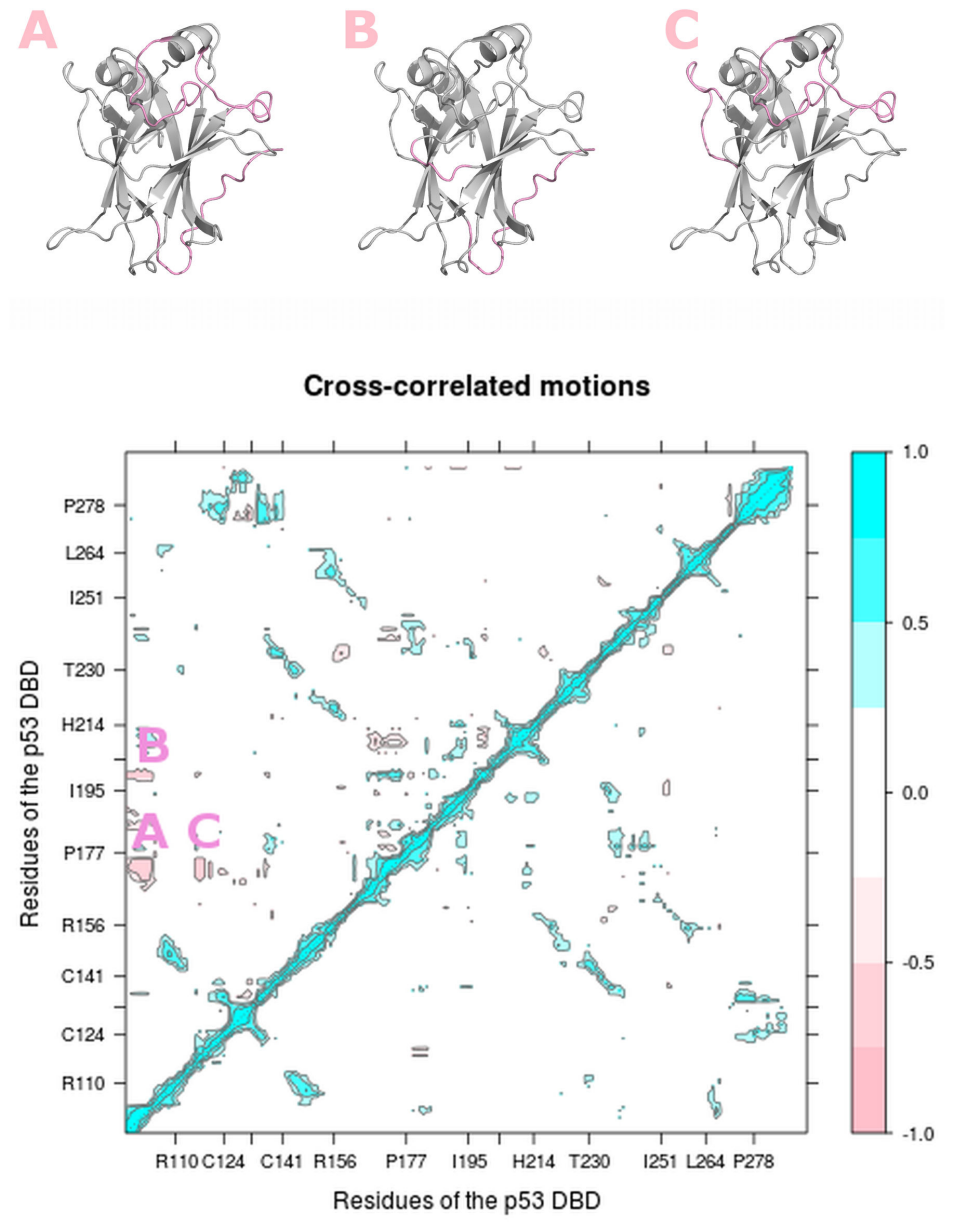
Cross-correlated motions of p53 DNA binding domain. (Upper panel) The anti-correlated motions (A-C) that are present in extended L1 but absent in recessed L1 conformers are colored in pink on the p53 DBD structures. The orientations of p53 DBD structures are the same as in Figure 1C. (Lower panel) The map of cross-correlated motions of extended L1 conformers (in upper triangle) and recessed L1 conformers (in lower triangle) shows different patterns between particular regions. For clarity, only correlated motions with absolute normalized values of at least 0.25 are shown. Motion occurring along the same direction is represented by positive correlation (cyan), whereas motion occurring along opposite directions is represented by negative (anti-) correlation (pink).

The fluctuations of L2 are higher than those of L3 (Figure 5). The N-terminal residues are highly flexible as expected. The C-terminal residues are less flexible than the N-terminal residues probably because of the presence of an α helix (H2) at the C-terminal end of our simulated structures. The high flexibility of H2 (residues P278-E287) relative to H1 possibly arises as it is located at the C-terminal. Secondly, H2, together with dominant L1, plays roles in the binding of DBD to the major groove of DNA. Thirdly, H2 is spatially located near L1 and hence they are likely to engage in short-range 'neighbor' communications. Fourth, a simulation study of the oncogenic mutant R282W p53 DBD (the residue 282 is on H2) suggested that the mutation correlates with backbone deviations not only of H2 but also of the S7-S8 loop (residues Y220-C229) [66]. The S7-S8 loop is distant from H2, hence their result infers the possibility of long-range communication between the H2 and S7-S8 loop in the p53 R282W mutant DBD, which we do not see in our wild-type simulations (Figure 6). This observation demonstrates that although both wild type and R282 p53 DBDs have similar structures, the dynamics of the two are likely to be significantly different. 

**Figure 7:**
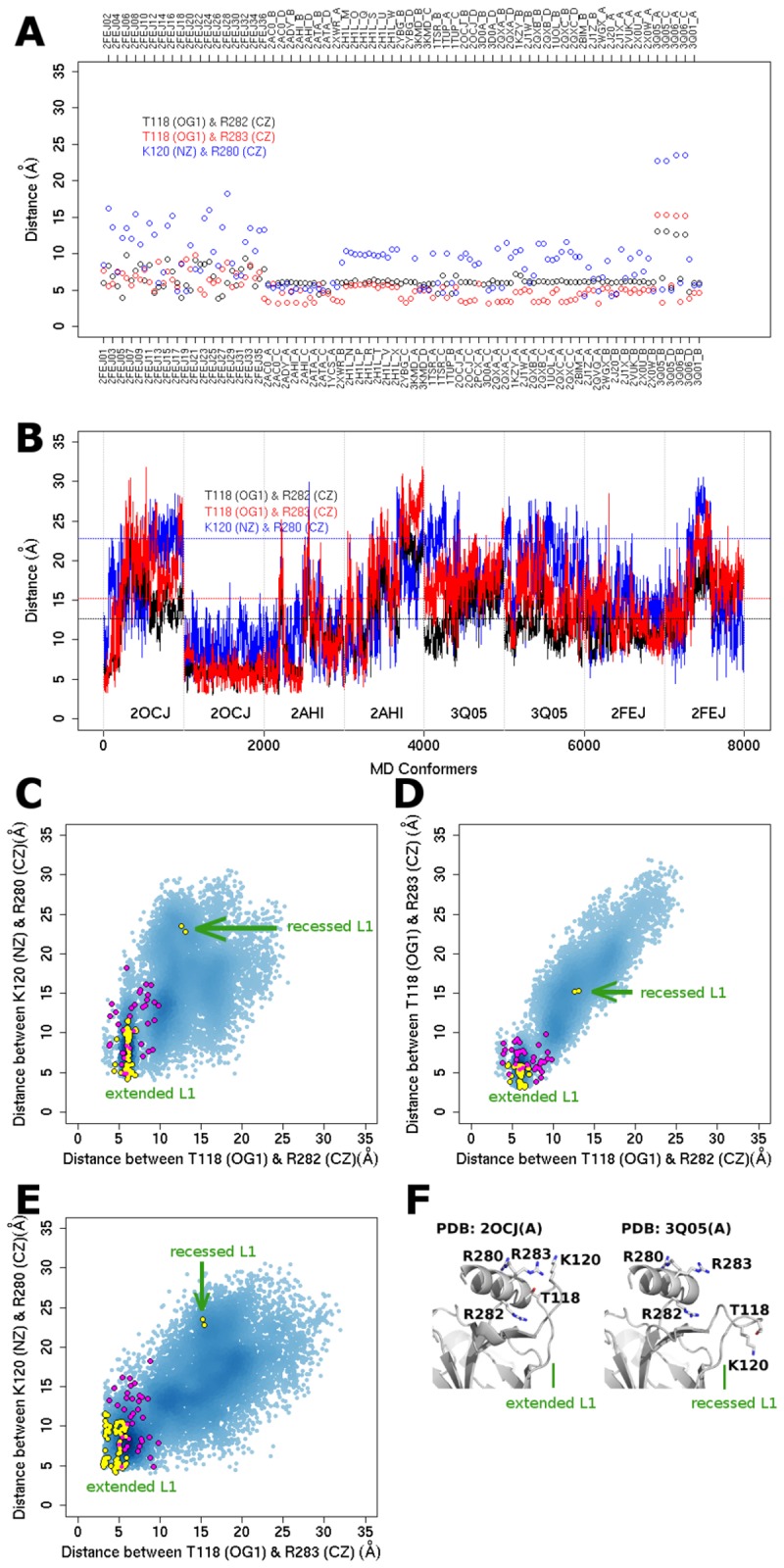
L1 and H2 are distant to each other in recessed L1 conformers. (A) The profile of the distances between residues in L1 and H2 that increase in recessed L1 conformers, across experimentally determined structures. (B) The profile of the distances indicated in (A) across MD conformers analyzed in this study. Horizontal dotted lines indicate the threshold for the distances in experimentally determined structures with recessed L1 conformations. Vertical dotted lines demarcate independent MD simulations started from four different starting structures, each with two copies of different initial velocity. (C-E) Reaction coordinates to characterize distinct L1 conformations, where recessed L1 conformational space is sampled by MD conformers (blue). Crystal and NMR structures are colored in yellow and magenta respectively. (F) Residues T118, K120, R280-R283 are shown as sticks.

To investigate the relationship between L1 and H2 because of possible short-range 'neighbor' communications between them, we analyze the distances between representative residues in L1 and those in H2 (Figure 7). The representative residues were identified based on changes in distances between pairs of residues in the experimental structures with recessed and extended L1 conformations (Figure 7A). The distributions of the distances between three pairs of representative residues (T118 and R282, T118 and R283, K120 and R280 in Figure 7F) separate the extended and recessed L1 of experimental structures (Figure 7C-E). Moreover, MD simulations of wild-type p53 DBD (except the 2^nd^ copy of a simulation started from the crystal structure with PDB code 2OCJ) can sample the recessed L1 conformational space (Figure 7B-E), highlighting the intrinsic dynamics of L1 in sampling both extended and recessed L1 conformations, even in the absence of DNA. It appears that L1 and H2 move apart in the recessed L1 conformational states (Figure 1B, Figure 7F). 

Residue R280 of H2 makes direct contact with DNA regardless of the conformational states of L1 [17,38,40]. This positioning of R280 of H2 hence allows the measurement of its separation from K120 of L1 as a feature that distinguishes the extended from the recessed L1 conformations. Another residue of H2, R282 plays a stabilizing role by linking L1 (Figure 7F); R282 is among the top seven p53 DBD hot spot residues with the highest mutation rates in cancers [5]. It has been demonstrated that in the R282W mutant, L1 undergoes enhanced flexibility [67]. The tryptophan results in steric hindrance, the loss of a critical hydrogen bond (between R282 and T125 in the wild type), and the consequent loss of the hydrogen bond network that stabilizes L1, as evidenced by the inconclusive electron density of residues S117-S121 in the crystal structure of R282W (PDB code 2J21) [68]. In contrast, in the R282Q mutant, L1 has been reported to have reduced flexibility [69]. Q282 engages in a new hydrogen bond with S116. As a result of this hydrogen bond, H115 in L1 possibly forms two novel hydrogen bonds with G117 and C124; these hydrogen bonds are absent in the wild type structure. Together it is hypothesized that these three new hydrogen bonds lead to reduced flexibility of L1. These contrasting effects of R282W and R282Q mutations highlight the role that hydrogen bond networks play in modulating the dynamical influence of H2 on L1. We next quantify the relationship between the dynamics of L1 and H2 through correlated dynamics analysis (see next section).

### Differences between extended and recessed L1 dynamics

A number of studies have indirectly shown that L1 is very flexible. In the p53 DBD dimer-DNA complex (PDB code 2GEQ), L1 is disordered [19]. To complement the previous studies, our simulations show a highly flexible L1, adopting both the extended and recessed conformational states. The crystal structure of worm p53 homologue, CEP-1, has a recessed L1 conformation (PDB code 1T4W) [70]. The authors proposed that this results from a helical region within L1 that repositions the entire L1. In the ensemble of crystal structures analyzed in this study, we detect no helix in L1 (using both Stride and DSSP). However, in the ensemble of NMR conformers, DSSP (but not Stride) suggests the presence of a 3_10_ helix (residues A119-S121) in L1 in six NMR conformers (out of a total of 36 NMR conformers). We also observed the transient helix in L1 in 4.9 % of the wild type MD conformers as determined using DSSP (Figure S14). In the MD conformers, the transient helix is formed by residues A119-S121 or S117-A119. The helix A119-S121 appears to be stabilized by residues S269 and F270 of S10, whereas the helix S117-A119 interacts with residues L111, G112 of S1, H115 of L1, and S269, F270 of S10. Besides the intermolecular contacts, K120 of the transient helix has a large solvent accessible surface area of ~178 Å^2^, suggesting that solvent plays a role in stabilizing the helix through hydrogen bonds.

We discover that isolated (DNA-free) monomeric p53 DBD can intrinsically sample both extended and recessed L1 conformations in MD simulations. In simulations initiated from the crystal and NMR structures with extended L1, MD conformers can adopt L1 conformations that are recessed, as evidenced by the smaller RMSD with respect to recessed L1 than the RMSD with respect to extended L1 (Figure S15). Again, in MD simulations initiated from a crystal structure with recessed L1, its MD conformers can also adopt L1 conformations that are extended (Figure S15 and Supplementary Information in File S1). This observation gives rises to the following questions. What are the differences between both L1 conformations? Does the difference in the L1 region affect other regions in p53 DBD? 

To address these questions, we classified available MD conformers (of the cumulative 0.8 μs simulation time) into either extended L1 or recessed L1 states based on the stringent criteria that the L1 RMSD ≤ 2 Å with respect to crystal structures with extended L1 (PDB code 2AHI, 2OCJ, 2FEJ) or recessed L1 (PDB code 3Q05) respectively. The extended L1 dataset has 3113 MD conformers, whereas the recessed L1 dataset has 357 MD conformers. 

The flexibility of L1, L2, the S5-S6 loop and the S6-S7 loop is higher in MD conformers with extended L1 than in those with recessed L1 (Figure 5). In terms of secondary structure composition of L1, our simulation results seem to support the presence of a helical region within recessed L1 as was reported for CEP-1 [70]. According to DSSP and Stride, 43.4% and 7.6%, respectively of recessed L1 conformers generated from molecular dynamics simulations contain a helix in L1. This helix introduces a rigidity in the loop region that probably leads to the attenuated flexibility. 

To further understand the distinct dynamics between extended and recessed L1 conformers, we analyzed their correlated motions by calculating the dynamic cross-correlation matrix (DCCM) [71]. DCCM has been successfully used to identify correlated motions between pairs of regions in diverse proteins such as bovine pancreatic trypsin inhibitor [71], Ras [34] and titin immunoglobulin domains [72]. We normalized the correlation values to range from -1 to 1. Positive correlation values represent correlated motions in which the residues move along the same direction, whereas negative values represent motions occurring in the opposite direction. Values close to zero indicate that the motion is uncorrelated. 

In both extended and recessed L1 conformers, the following regions have strong correlated motions: between S1 and S3, S2’ and the S10-H2 loop, S3 and S8, H1 and L3, S5 and S8 regions (Figure 1c and Figure 6). The correlated motion between H1 and L3 is expected because both H1 and L3 are in close proximity. In contrast, the following anti-correlated motions are present only in the extended L1 conformers: between N-terminal and L2, N-terminal and the S5-S6 loop, L1 and L2 (Figure 6). 

**Figure 8:**
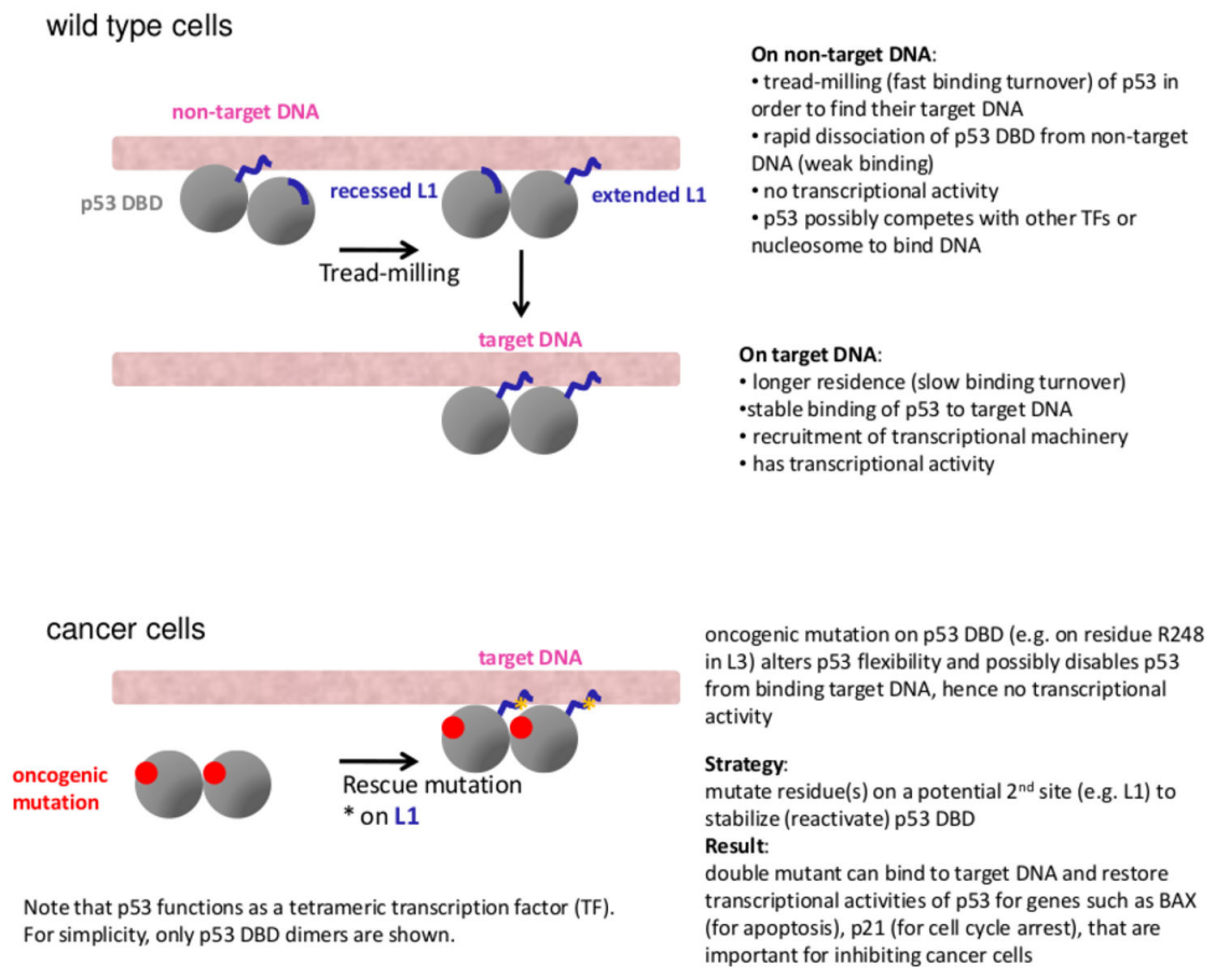
The possible roles of conformational dynamics of L1 in tread-milling and stable binding of p53 to non-target and target DNA binding sites, respectively. In cancer cells, oncogenic mutants on p53 DBD can possibly be rescued with the 2nd site mutation on L1 through lever-like flexibility re-distribution.

The absence of the anti-correlated motion between L1 and L2 in the recessed L1 states may arise from the increasing separation between L1 and H2 (Figure 7D), where H2 is situated in between L1 and L2 (Figure 1B), and the loss of a stable hydrogen bond network (Figure S16B, discussed in the next paragraph). Interestingly, the anti-correlated motion between L1 and L2 that is seen only in the extended L1 states suggests the following hypothesis: in order to bind DNA at the major groove, L1 is likely to adopt an extended conformation (Figure 8), while L2 binds the minor groove. L1 and L2 are known to bind to major and minor groove respectively. Therefore, the long-range negative correlation between the two loops (separated by 24.3 Å) may play a significant role in enabling the two loops to search for their binding sites on DNA and optimally embed into the two grooves.

Next, we compare hydrogen bonds present in p53 DBD MD conformers with extended versus recessed L1. In the recessed state, L1 forms an average of 8 hydrogen bonds, whereas extended L1 forms an average of 5 hydrogen bonds. The total number of hydrogen bonds appears to be similar for both p53 DBD with extended and recessed L1 (Figure S16A), with a slightly higher number of hydrogen bonds (based on 20-80% occupancy) in the DBD with recessed L1 than the DBD with extended L1. However, some highly stable hydrogen bonds (based on 90% occupancy) are present in DBD conformers with extended L1 but absent in those with recessed L1. Based on 90% occupancy, six hydrogen bonds that are present in MD conformers with extended L1 (Figure S16B), are lost in MD conformers with recessed L1, for example a hydrogen bond between an S2 residue T125 (atom OG1) and an H2 residue R282 (atoms HH11 and NH1). Four of these hydrogen bonds are backbone hydrogen bonds, and two are side chain hydrogen bonds. In addition to the hydrogen bonds that are present in both types of MD conformers with extended and recessed L1, the network of these six highly stable hydrogen bonds are likely to be important in the dynamics of p53 DBD with extended L1, because the residues involved in the hydrogen bonds, as members of the network, appear to contribute to long-range communication between distant sites, for example between L2 and H2 (Figure S16C).

**Figure 9:**
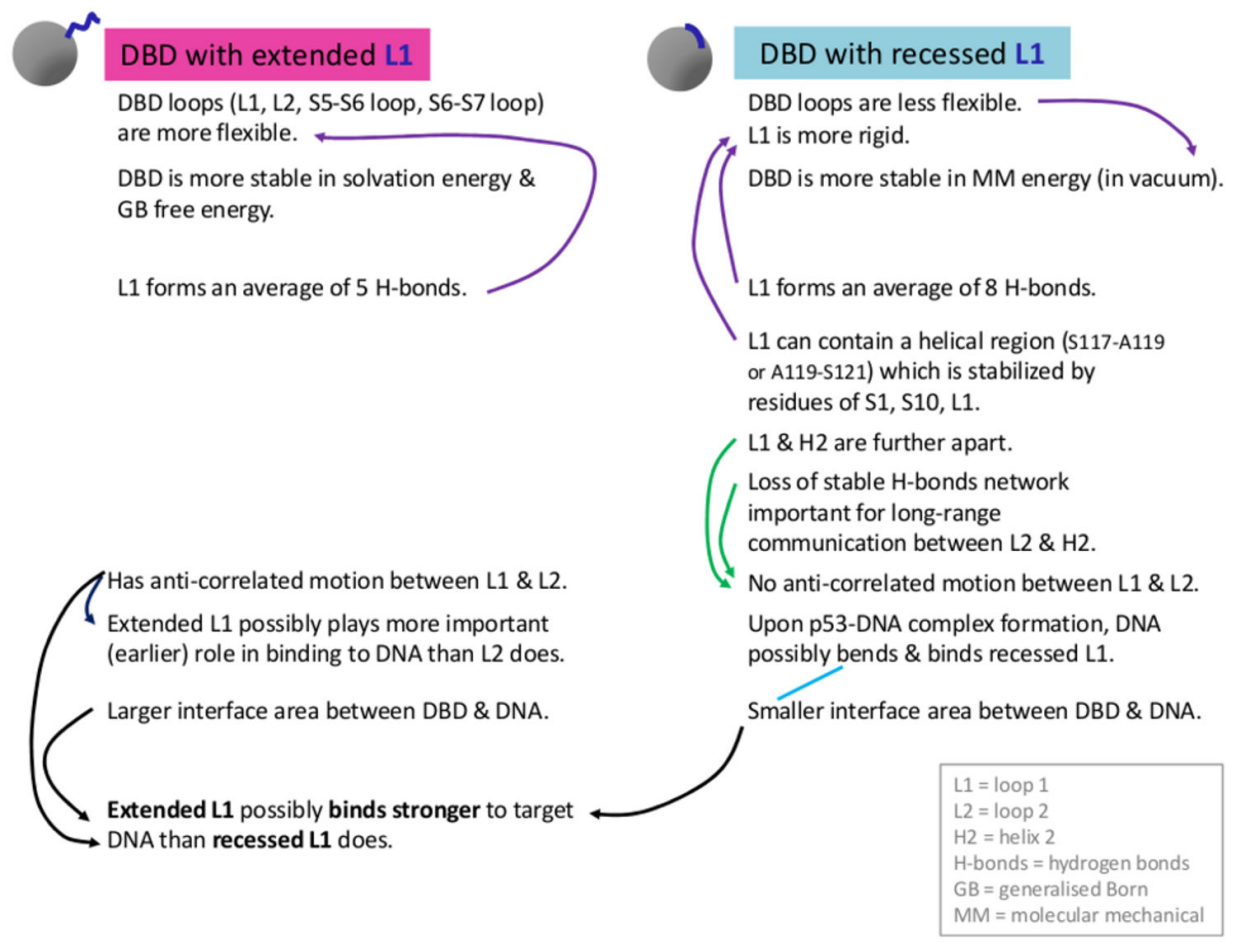
Comparisons between the different dynamics of DBD with extended and recessed L1 conformations to propose their possible distinct involvement in binding to DNA.

Given these differences, we ask why in a single complex of DNA and p53 DBD tetramer such as in PDB code 3Q05 [38] and 3TS8 [39], the DBD can adopt both extended and recessed L1 states. If extended L1 enables a better initial binding of DBD to DNA, then the subsequent DBD (even with recessed L1) will possibly find it easier to bind in the context of cooperative binding, which is known to characterize p53-DNA binding [60,73–75]; we discuss this in detail in the Supplementary Information in File S1 and Table S1. In brief, upon forming a complex with p53 DBD with extended L1 conformation, DNA bends and binds recessed L1 conformation without steric clashes [39]. Moreover, it is possible that the diversity of L1 conformations facilitate the p53 DBD to search for their target DNA through tread-milling [64,65] and to bind stably once it locates its target DNA (Figure 8); this process seems possible through the regulation of binding off rates against non-specific versus specific (target) DNA [38,39]. In addition, the average interface area between DNA and monomeric DBD with extended L1 (500.3Å^2^) is larger than with recessed L1 (490.6 Å^2^) as calculated using NACCESS [76]. The bending of DNA observed in the interface with DBD adopting recessed L1 (PDB codes 3Q05, 3Q06, 3TS8) possibly compensates for the smaller interface area. This difference in the interface area for monomeric p53 DBD-DNA suggests that extended L1 possibly contributes to stronger binding of DBD to DNA than recessed L1 does. Herein, we summarize the different structural dynamical behaviors of p53 DBDs with extended and recessed L1 (Figure 9), and highlight their possible distinct involvements in binding to DNA (Figure 8, Figure 9). 

### L1 dynamics is important for p53 function and can potentially rescue loss of function

L1 is important for binding to DNA, turning on trans-activation and apoptosis based on results from yeast-based assays [77,78], cell-based assays [79,80] and cell-free assays [81]. Our simulations shed new light on the atomistic details of L1 dynamics (at timescales inaccessible to experiments) and the potential implications for p53 functions. 

L1 (residues F113-T123) is a mutational cold spot [5], as opposed to mutational hot spot residues (such as Y220 in the S7-S8 loop, G245 and R248 in L3), with relatively few cancer-related mutations identified in L1. A mutagenesis study showed that both K120A and T123A mutants of p53 DBD were capable of binding DNA better than the wild type did *in vitro* [79]. S121F mutant also binds stronger to some p53-binding sites on DNA and is able to induce apoptosis better than the wild-type does [80]. T123A mutant also binds stronger to p21 promoters and induces greater apoptosis than the wild type does [81]. It seems that mutations in L1 have the opposite effects from the cancer-related mutations. Furthermore, a second-site mutation on L1, H115N, can rescue the primary mutation on L3, R248Q [31]. In wild type p53 DBD, four hydrogen bonds anchor R248 on L3 to the DNA minor groove [17]. The substitution of Arg to Gln likely destabilizes the hydrogen bond network [32], and the destabilization may in turn increase the flexibility of L3. This hypothesis is substantiated by a simulation study showing that reduced flexibility (rigidification) due to a mutation on L1 (H115N) can compensate for the enhanced flexibility due to a mutation on L3 (R248Q) [31]. Such a mechanism of lever-like redistribution of flexibility is present in other proteins, such as PDZ domain [82], dihydrofolate reductase [83], and actin capping protein [84]. 

**Figure 10:**
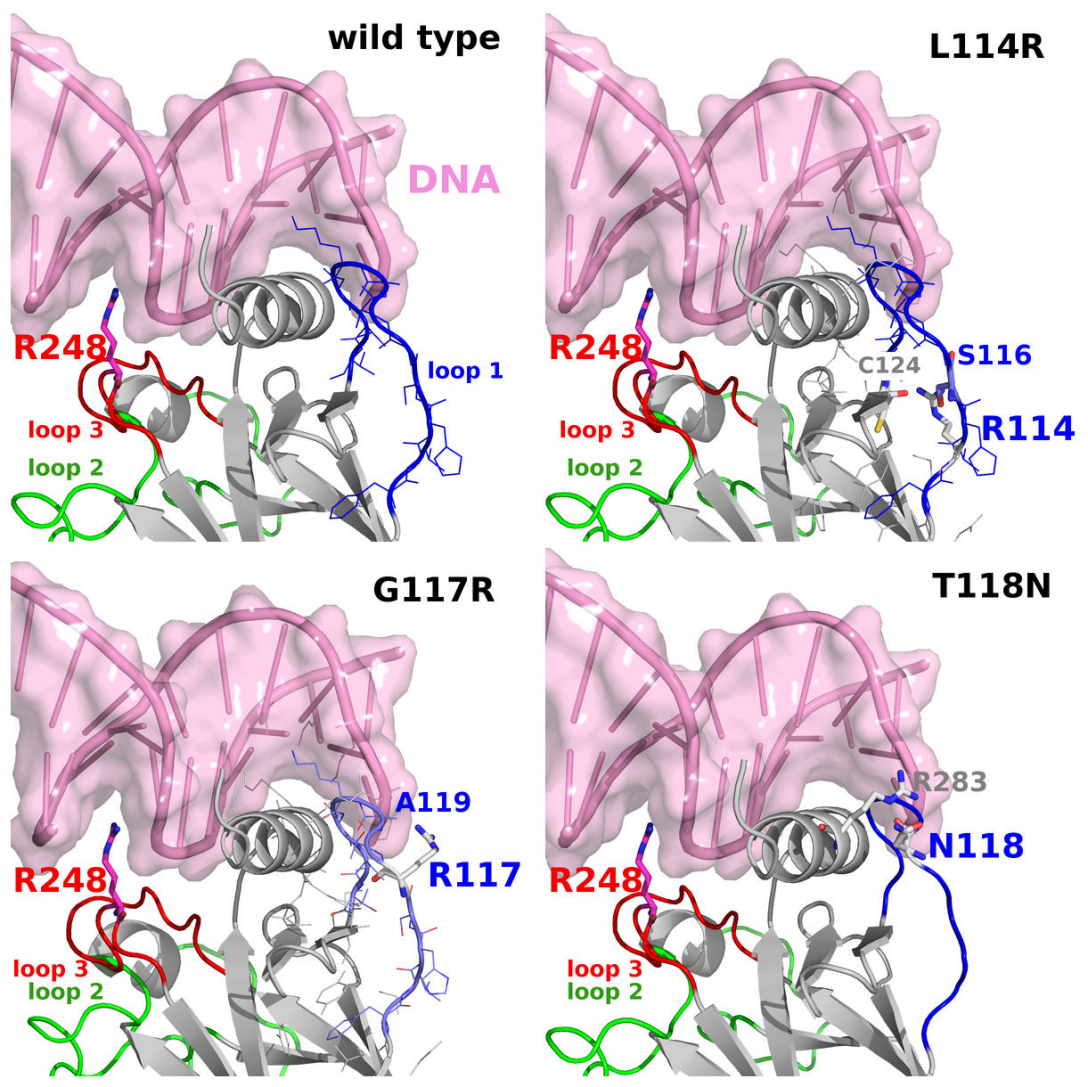
The proposed rescue mutants of L1 for the oncogenic R248Q, whose L3 experiences enhanced flexibility due to the mutation. In the L114R mutant, R114 forms hydrogen bonds with S116 of L1 and C124. In the G117R mutant, R117 forms a hydrogen bonds with A119. In the T118N mutant, N118 forms a hydrogen bond with R283. In each panel, L1, L2, L3 and DNA are colored in blue, green, red and pink, respectively.

Why is it that the wild-type p53 DBD has flexible L1 that binds DNA well but not as well as the S121F and T123A mutants do? A recent work based on the transcription factor Rap1 [64] suggests that transcription factors, in general, tread-mill on DNA (Figure 8) in order to find their target DNA binding site. If the DNA sequence is not the target, the transcription factor will dissociate very rapidly. Moreover, distinct target DNA sequences have been suggested to have different off-rates for p53 DBD [38,39]. It is possible that the high flexibility of L1 of p53 DBD plays an important role in this process of tread-milling (Figure 8). Another study on DNA polymerase β shows that its lyase domain is flexible in the DNA-free state, but is dominantly quenched in its motions when bound to DNA [85]. On the other hand, its polymerase domain is less flexible in the DNA-bound state than in the free state. Together these suggest that L1 of p53 DBD can be a promising druggable site in order to modulate the effects of oncogenic mutations. Based on visual inspections, we propose L114R, G117R, and T118N (Figure 10) as possible mutations in L1 that will likely attenuate the flexibility of L1 and, by long-range communications, that of L3 carrying the oncogenic R248Q mutation. 

There exists a complex relationship between protein flexibility and stability [30,57,86,87]. Increases in flexibility can, but does not necessarily always result in a decrease in stability. It is important to consider intrinsic stability (contributed by the molecular mechanical energy) and the effect of solvation (Figure 9, Figure S12). Wild type p53 has not naturally evolved to maximum stability as is evident from the fact that it still has unsatisfied hydrogen bond donors and acceptors [7]. Proteins like p53 are not very stable, and this instability may be related to the regulations of their levels [88] and locations in cells. This relationship between flexibility and stability can possibly be one of the explanations for the superior DNA binding affinity of S121F and T123A relative to the wild-type p53 DBD. 

In conclusion, through multiple copies of MD simulations of the p53 DBD, this study (i) shows that the structure of p53 DBD is substantially more dynamic than what has been apparent from crystal structures *per se*, (ii) analyzes the differences in dynamical behaviors of recessed and extended L1 conformational states, and proposes their distinct involvements in binding to DNA, and (iii) highlights how the dynamics of L1 provides a promising avenue for therapeutic interventions through long-range (allosteric) modulations. Such efforts have been given an optimistic boost recently by the computer aided discovery of a small molecule that re-stabilizes a mutant form of p53 [89]. 

## Materials and Methods

### Analysis of the crystal and NMR ensemble

We retrieved homologous structures to p53 DBD (PDB code 2OCJ) from the RCSB PDB and performed bioinformatics-based principal component analysis (PCA) using the Bio3D package of R [36]. 

To identify core positions, iterated rounds of structural superposition were used to identify the most structurally invariant region of the p53 DBD structure. This procedure, implemented in the Bio3D package, entailed excluding those residues with the largest positional differences, before each round of superposition, until only the invariant core residues remained. We then used the structurally invariant core as the reference frame for structural alignment of crystal, NMR and simulation conformers (structures). The simulation conformers were obtained from performing molecular dynamics (MD) simulations.

Next, we used the Cartesian coordinates of aligned Cα atoms to define the elements of a covariance matrix. The covariance matrix was then diagonalized to derive principal components (PCs) with their associated variances. PCA enables the projection of crystal, NMR and MD conformers on the subspace defined by PCs with the largest variances. Such an approach has been successfully employed to study the conformational diversity in the Ras [34,90] and HIV-1 protease [37] proteins. Secondary structures were assigned using both DSSP [91] and STRIDE [92].

### Molecular dynamics (MD) simulations

Systems for molecular dynamics (MD) simulations were prepared from high-resolution crystal structures and a model of NMR structure of wild-type p53 DBD. In the original crystal structures, the p53 DBD structure is DNA-bound in the PDB code 2AHI [17] and 3Q05 [28], whereas the p53 DBD is DNA-free in the PDB code 2OCJ [18]. Their resolutions are 1.85, 2.40 and 2.05 Å, respectively. In the NMR structure (PDB code 2FEJ model / conformer 11) [7], the p53 DBD structure is DNA-free. Conformer 11 was chosen because it has the lowest RMSD to other NMR conformers. 

We extracted the monomeric p53 DBD structure, together with the Zn^2+^ ion, from each entry to perform dual copies MD simulations for each starting structure, by varying the initial velocities. Therefore, we have eight independently simulated systems (and hence trajectories of 100 ns each) to enhance the sampled conformational space. The total simulation time for the combined trajectories is 0.8 microseconds.

Each system was simulated using the ff99SB force field [93] in the AMBER 11 package [94]. The LEaP module of AMBER was used for adding missing atoms to the initial coordinates, including the parameters for the Zn^2+^ ion, neutralizing the systems with charge neutralizing counter ions at pH 7, and adding TIP3P water molecules [95] with the buffering distance set to 12 Å . The protonation states for all titratable residues were determined using PDB2PQR [96]. The systems were neutralized to attain a total net charge of zero in order to effectively treat electrostatic interactions using Particle Mesh Ewald (PME) summation [97]. 

Energy minimization with decreasing constraints on the positions of heavy atoms was initially performed for 1000 steps. Constant volume heating (to 300 K) was carried out for 50 ps. Next, equilibration at constant temperature (300 K) and constant pressure (1 atm) was performed for 2 ns.

The NPT (isobaric–isothermal) ensemble at 300 K, 1 atm, and long-range non-bonded interactions with a 10 Å atom-based cutoff were used for the production simulations. The temperature was maintained using Langevin dynamics[98] with a collision frequency of 1 ps^-1^ and the pressure was maintained using weak-coupling with a pressure relaxation time of 1 ps. The SHAKE algorithm [99] was used to constrain all covalent bonds involving hydrogen atoms, therefore enabling a longer time step of 2 fs. 

Trajectory analyses were performed using the *ptraj* module of AMBER, and the Bio3D package of R [36]. The time evolution of the structures, as measured by RMSD (Figure S3), suggest that the simulations were stable, in particular for the core of the p53 DBD structures. The binding energies between proteins were computed using the molecular mechanics/Generalized Born surface area (MM-GBSA)[100,101] approximation using the GB module in Amber while the non-polar component was estimated from the solvent accessible surface area using MOLSURF [102] with ΔG_solv,np_ = 0.00542*SASA +0.92 [103].

### Normal mode analysis

We performed coarse-grained AD-ENM normal mode analysis [54], that uses a single-parameter Hookean potential, that has previously been shown to give low-frequency normal modes that are agreeable with modes obtained from empirical force [55].

## Supporting Information

Figure S1 **The residual contribution of PC 1 to PC 3 reflects the dynamics of individual residues.** (Left panel) Secondary structures of alpha helices and beta sheets are represented as black and grey boxes on horizontal axes. (Right panel) For each PC, equidistant atomic displacements from the mean structure are mapped onto the structure of p53 DNA binding domain. The N- and C-terminal of p53 DBD are labeled in cyan and magenta, respectively. The important loops in the p53 DBD: loop 1, loop 2 and loop 3 are colored blue, green and red, respectively.
(TIFF)

Figure S2 **The relationships between PC 1 and (**A**) surface area (**B**) volume, and (**C**) radius of gyration.** Crystallographic and NMR conformers are colored in yellow and magenta, respectively.
(TIFF)

Figure S3 **The Cα RMSD with respect to the first conformer as a function of simulation time of wild-type p53 DBD started from the crystal structures of 2OCJ, 2AHI, 3Q05, 2FEJ.** Different copies indicate different initial velocities in the beginning of simulation in order to allow enhanced conformational sampling. Black lines indicate all Cα atoms, grey lines indicate the Cα atoms of core residues.
(TIFF)

Figures S4 **Projection of MD conformers (diamonds) onto the first three PCs defined by the experimentally-determined crystal (yellow circles) and NMR (magenta circles) structures (see Figure 2).** The MD conformers are color mapped based on simulation time from 0 to 100 ns. The PDB code of starting structure used for each MD simulation is indicated on upper left.
(TIFF)

Figures S5 **Projection of MD conformers (diamonds) onto the first three PCs defined by the experimentally-determined crystal (yellow circles) and NMR (magenta circles) structures (see Figure 2).** The MD conformers are color mapped based on simulation time from 0 to 100 ns. The PDB code of starting structure used for each MD simulation is indicated on upper left.
(TIFF)

Figures S6 **Projection of MD conformers (diamonds) onto the first three PCs defined by the experimentally-determined crystal (yellow circles) and NMR (magenta circles) structures (see Figure 2).** The MD conformers are color mapped based on simulation time from 0 to 100 ns. The PDB code of starting structure used for each MD simulation is indicated on upper left.
(TIFF)

Figures S7 **Projection of MD conformers (diamonds) onto the first three PCs defined by the experimentally-determined crystal (yellow circles) and NMR (magenta circles) structures (see Figure 2).** The MD conformers are color mapped based on simulation time from 0 to 100 ns. The PDB code of starting structure used for each MD simulation is indicated on upper left.
(TIFF)

Figures S8 **Projection of MD conformers (diamonds) onto the first three PCs defined by the experimentally-determined crystal (yellow circles) and NMR (magenta circles) structures (see Figure 2).** The MD conformers are color mapped based on simulation time from 0 to 100 ns. The PDB code of starting structure used for each MD simulation is indicated on upper left.
(TIFF)

Figures S9 **Projection of MD conformers (diamonds) onto the first three PCs defined by the experimentally-determined crystal (yellow circles) and NMR (magenta circles) structures (see Figure 2).** The MD conformers are color mapped based on simulation time from 0 to 100 ns. The PDB code of starting structure used for each MD simulation is indicated on upper left.
(TIFF)

Figures S10 **Projection of MD conformers (diamonds) onto the first three PCs defined by the experimentally-determined crystal (yellow circles) and NMR (magenta circles) structures (see Figure 2).** The MD conformers are color mapped based on simulation time from 0 to 100 ns. The PDB code of starting structure used for each MD simulation is indicated on upper left.
(TIFF)

Figure S11 **The contacts between K120 of DNA-free DBD crystal structure and residues of 3 different crystal symmetry mates (in each panel).**
(TIFF)

Figure S12 **Comparison of energetic profiles of p53 DNA binding domain with extended and recessed loop 1.** The energetic distribution of (A) solvation energy (B) total molecular mechanics energy of p53 DBDs with extended and recessed L1. (C) The average and standard deviations of the energetic components. ELE is the non-bonded electrostatic energy. VDW is the non-bonded van der Waals energy. INT is the sum of bond, angle and dihedral energies. GAS is the sum of ELE, VDW and INT, i.e. the molecular mechanical energy in vacuum. GBSUR is the hydrophobic contribution to solvation free energy for generalized Born (GB) calculation.
(TIFF)

Figure S13 **None of NMR conformers (PDB code 2FEJ) adopts recessed L1 conformation, their L1 is colored in cyan.** For clarity, only L1 of NMR conformers is shown. Recessed L1 of PDB code 3Q05 chain A is colored blue. L2 and L3 are colored green and red, respectively.
(TIFF)

Figure S14 **The time-dependent secondary structure profiles of MD conformers, with α helices and β sheets shown in black and grey respectively, except that the transient helix present in L1 is shown in red.** The PDB code of starting structure used for each MD simulation is indicated on the top of each panel.
(TIFF)

Figure S15 **The RMSDs of L1 residues in MD conformers with respect to the L1 residues in the crystal structures with extended L1 (PDB code s 2OCJ and 2AHI in magenta and orange, respectively) and recessed L1 (PDB code 3Q05 in black).** The L1 RMSDs with respect to the L1 residues in the NMR structure (PDB code 2FEJ) are plotted in red. TS: Transitioning state between extended and recessed L1.
(TIFF)

Figure S16 **Hydrogen bonds in p53 DNA binding domain.** (A) The total number of hydrogen bonds in the p53 DBD MD conformers with extended L1 (magenta +) and recessed L1 (cyan x). (B) Stable hydrogen bonds that are present in MD conformers with extended L1 but are lost in MD conformers with recessed L1. (C) The mapping of residues involved in stable hydrogen bonds onto the crystal structure with extended L1 (PDB code 2OCJ). The secondary structure coloring is the same as in Figure 1C.
(TIFF)

Figure S17 **Visualization of dominant motions (gray) obtained from NMA of p53 DBD with extended (green) and recessed (cyan) loop 1.**
(TIF)

File S1 (DOC)

Table S1 (XLS)
