# Supplementary figures and images for: Mapping the Structural and Dynamical Features of Multiple p53 DNA Binding Domains: Insights into Loop 1 Intrinsic Dynamics
Source: PLoS One. 2013 Nov 12;8(11):e80221. doi: 10.1371/journal.pone.0080221 (PMC3855832; doi:10.1371/journal.pone.0080221)

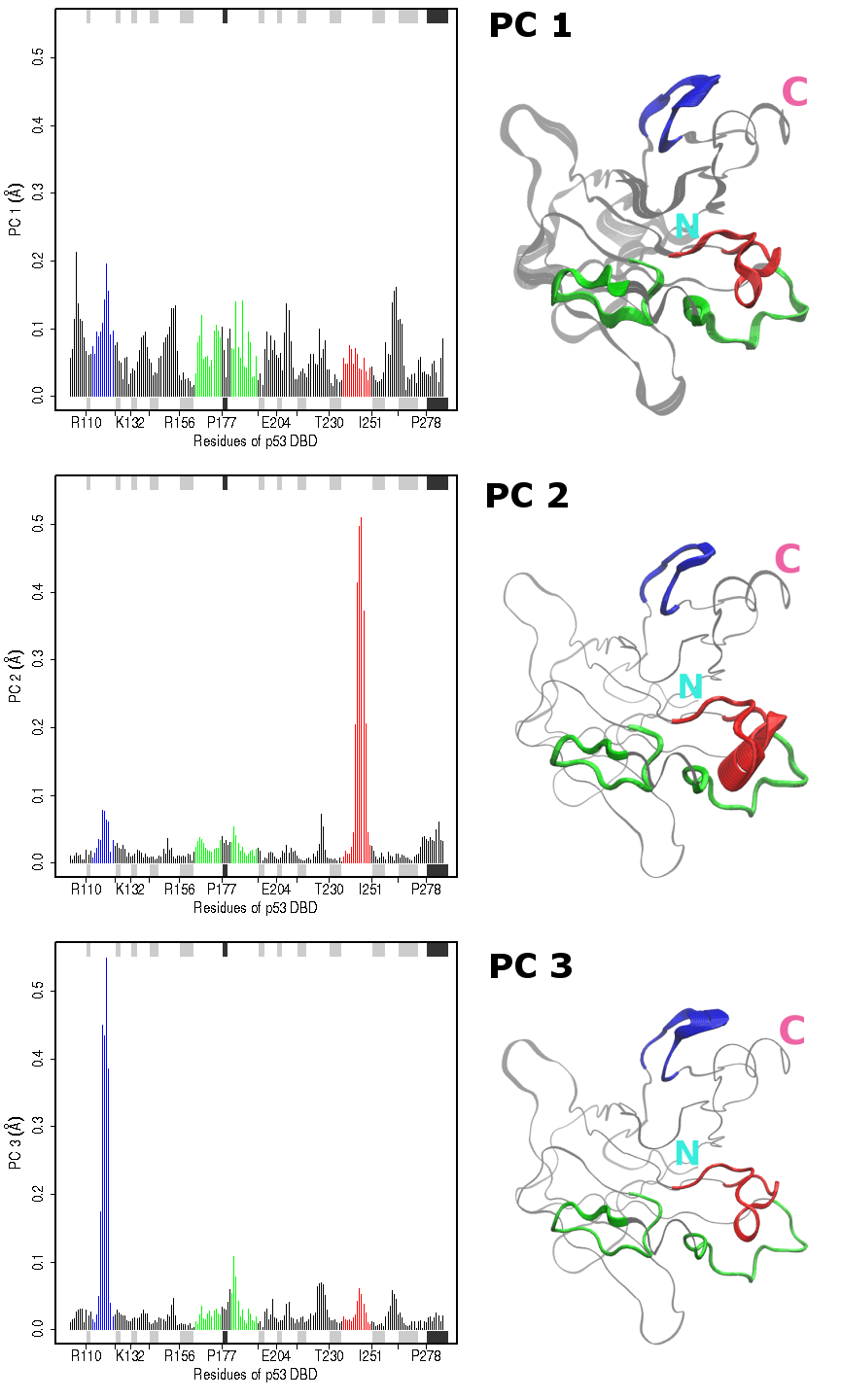

Supplement: Figure S1 — The residual contribution of PC 1 to PC 3 reflects the dynamics of individual residues. (Left panel) Secondary structures of alpha helices and beta sheets are represented as black and grey boxes on horizontal axes. (Right panel) For each PC, equidistant atomic displacements from the mean structure are mapped onto the structure of p53 DNA binding domain. The N- and C-terminal of p53 DBD are labeled in cyan and magenta, respectively. The important loops in the p53 DBD: loop 1, loop 2 and loop 3 are colored blue, green and red, respectively. (TIFF) [file pone.0080221.s001.tiff]

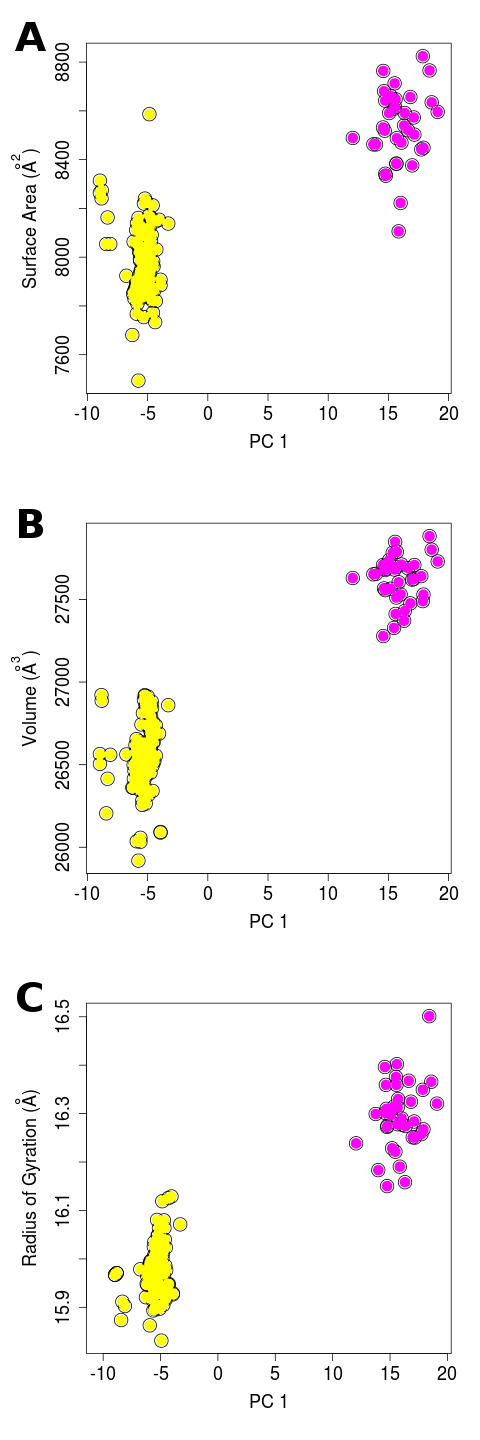

Supplement: Figure S2 — The relationships between PC 1 and (A) surface area (B) volume, and (C) radius of gyration. Crystallographic and NMR conformers are colored in yellow and magenta, respectively. (TIFF) [file pone.0080221.s002.tiff]

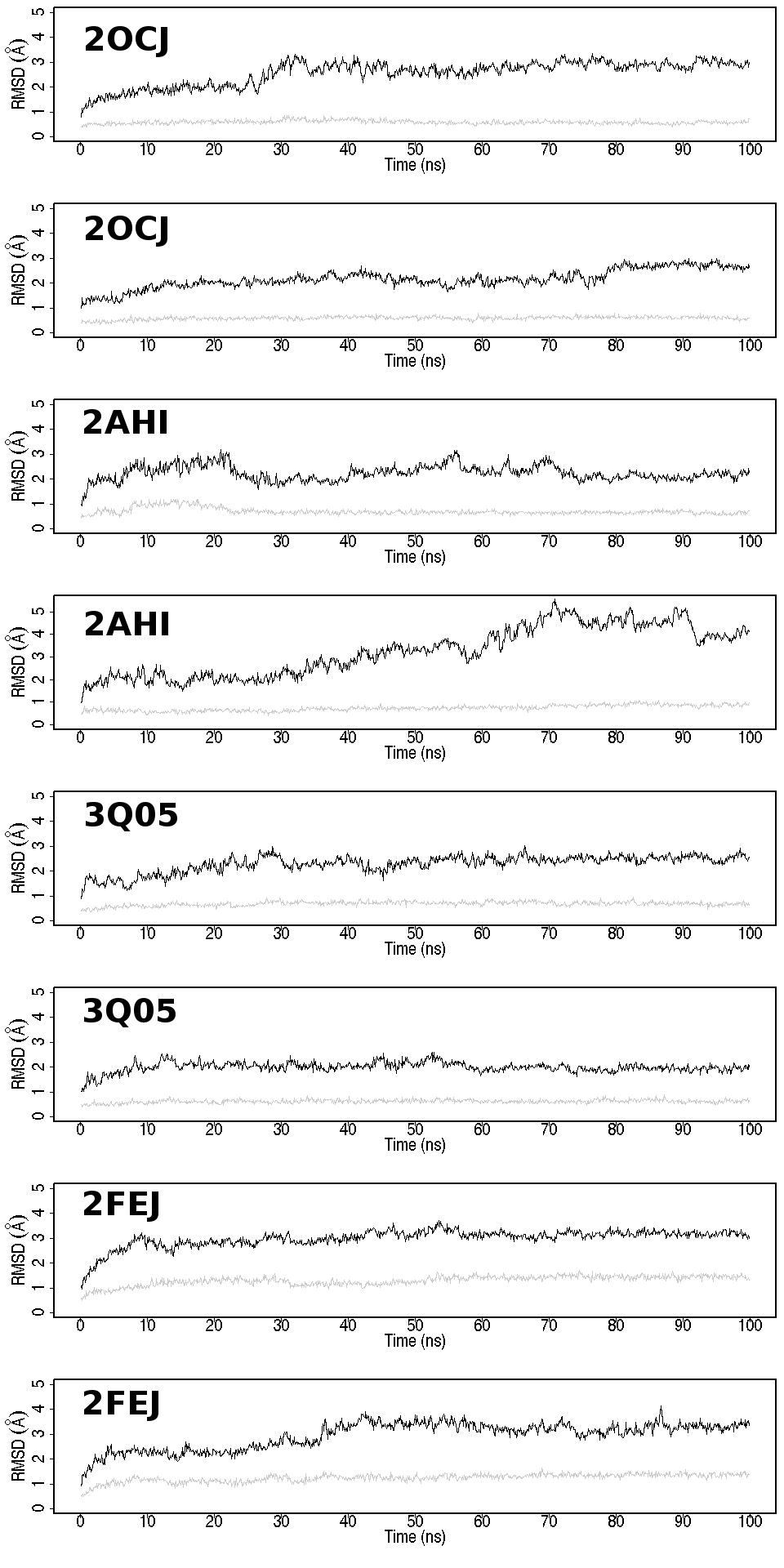

Supplement: Figure S3 — The Cα RMSD with respect to the first conformer as a function of simulation time of wild-type p53 DBD started from the crystal structures of 2OCJ, 2AHI, 3Q05, 2FEJ. Different copies indicate different initial velocities in the beginning of simulation in order to allow enhanced conformational sampling. Black lines indicate all Cα atoms, grey lines indicate the Cα atoms of core residues. (TIFF) [file pone.0080221.s003.tiff]

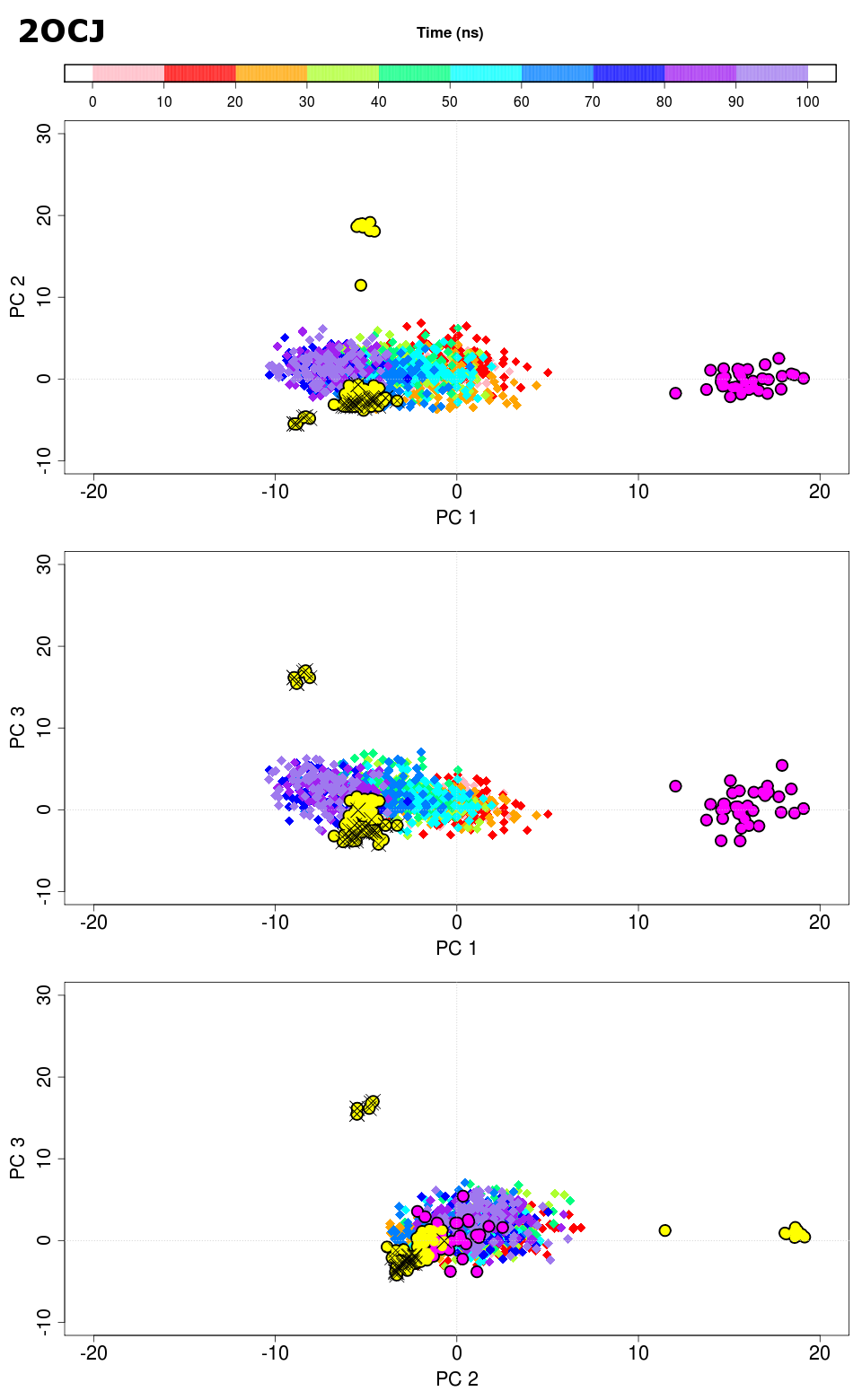

Supplement: Figures S4 — Projection of MD conformers (diamonds) onto the first three PCs defined by the experimentally-determined crystal (yellow circles) and NMR (magenta circles) structures (see Figure 2). The MD conformers are color mapped based on simulation time from 0 to 100 ns. The PDB code of starting structure used for each MD simulation is indicated on upper left. (TIFF) [file pone.0080221.s004.tiff]

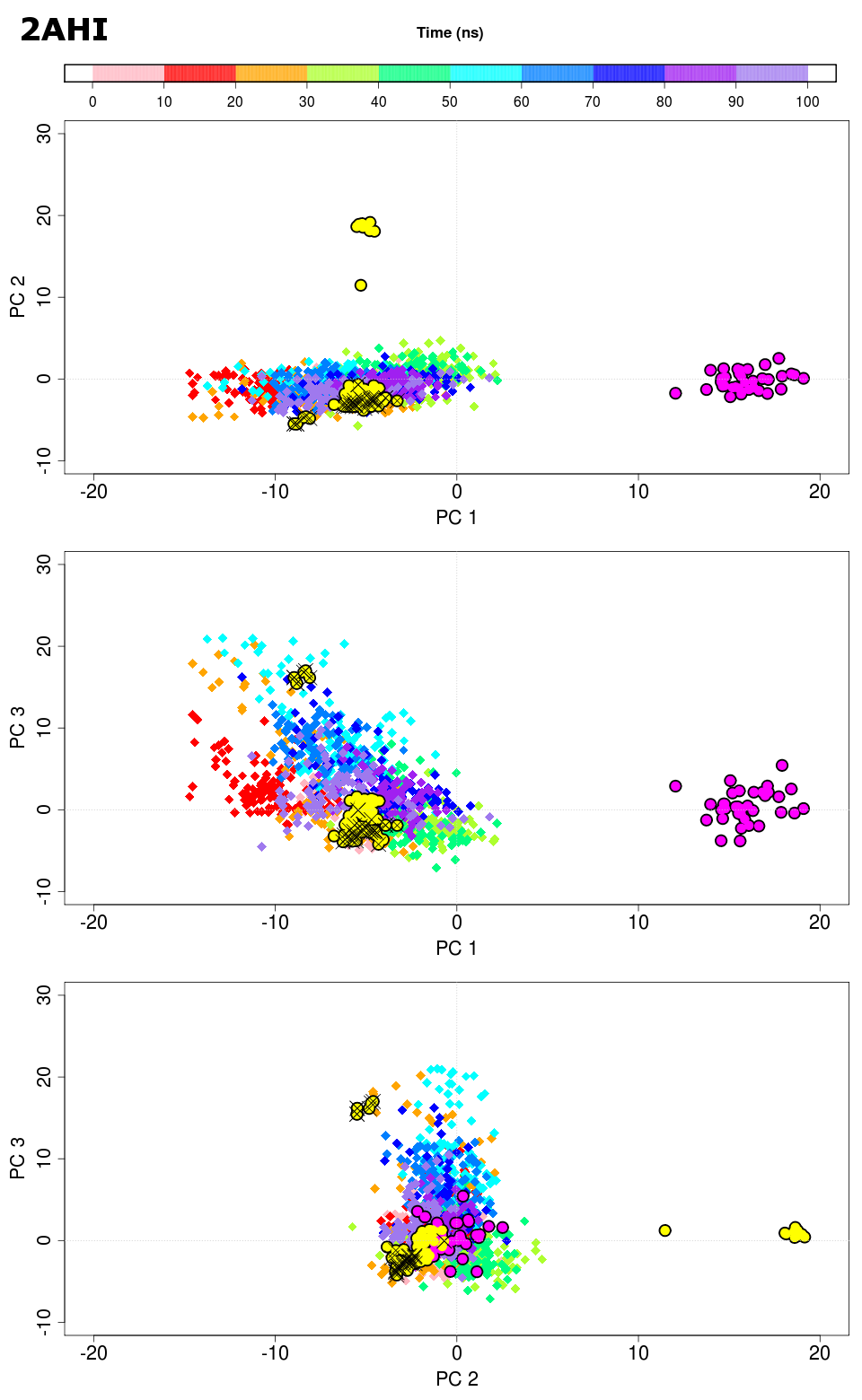

Supplement: Figures S5 — Projection of MD conformers (diamonds) onto the first three PCs defined by the experimentally-determined crystal (yellow circles) and NMR (magenta circles) structures (see Figure 2). The MD conformers are color mapped based on simulation time from 0 to 100 ns. The PDB code of starting structure used for each MD simulation is indicated on upper left. (TIFF) [file pone.0080221.s005.tiff]

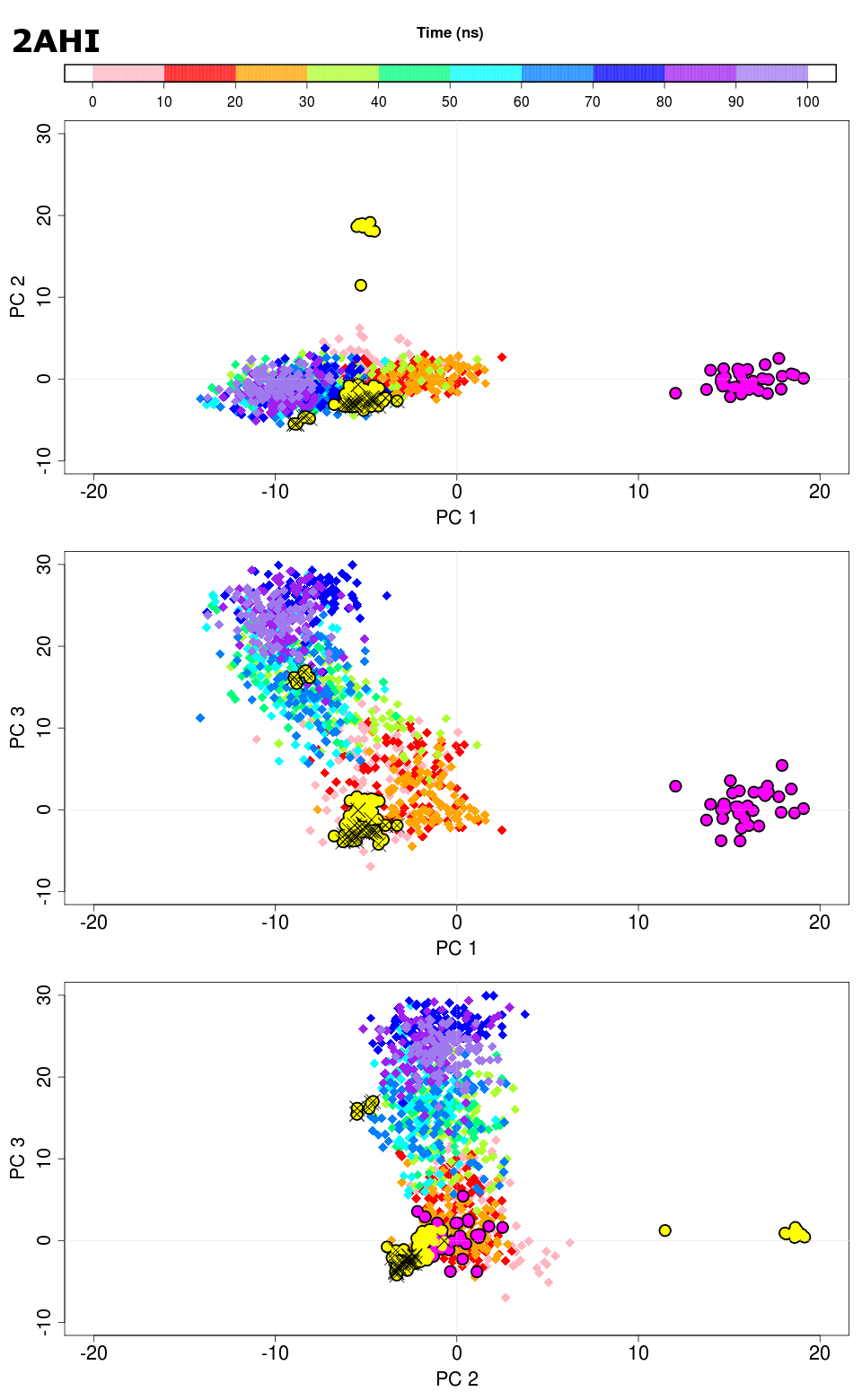

Supplement: Figures S6 — Projection of MD conformers (diamonds) onto the first three PCs defined by the experimentally-determined crystal (yellow circles) and NMR (magenta circles) structures (see Figure 2). The MD conformers are color mapped based on simulation time from 0 to 100 ns. The PDB code of starting structure used for each MD simulation is indicated on upper left. (TIFF) [file pone.0080221.s006.tiff]

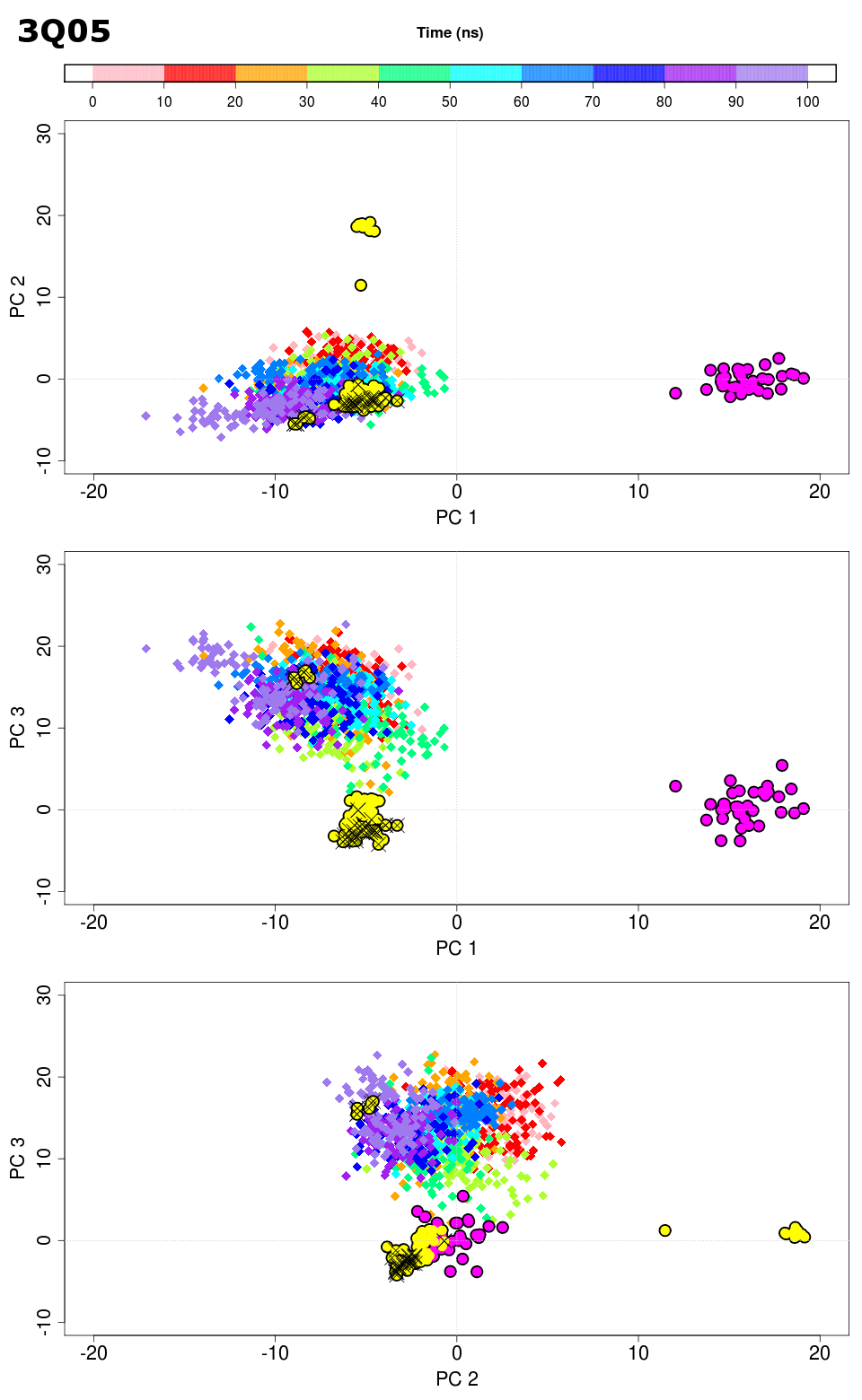

Supplement: Figures S7 — Projection of MD conformers (diamonds) onto the first three PCs defined by the experimentally-determined crystal (yellow circles) and NMR (magenta circles) structures (see Figure 2). The MD conformers are color mapped based on simulation time from 0 to 100 ns. The PDB code of starting structure used for each MD simulation is indicated on upper left. (TIFF) [file pone.0080221.s007.tiff]

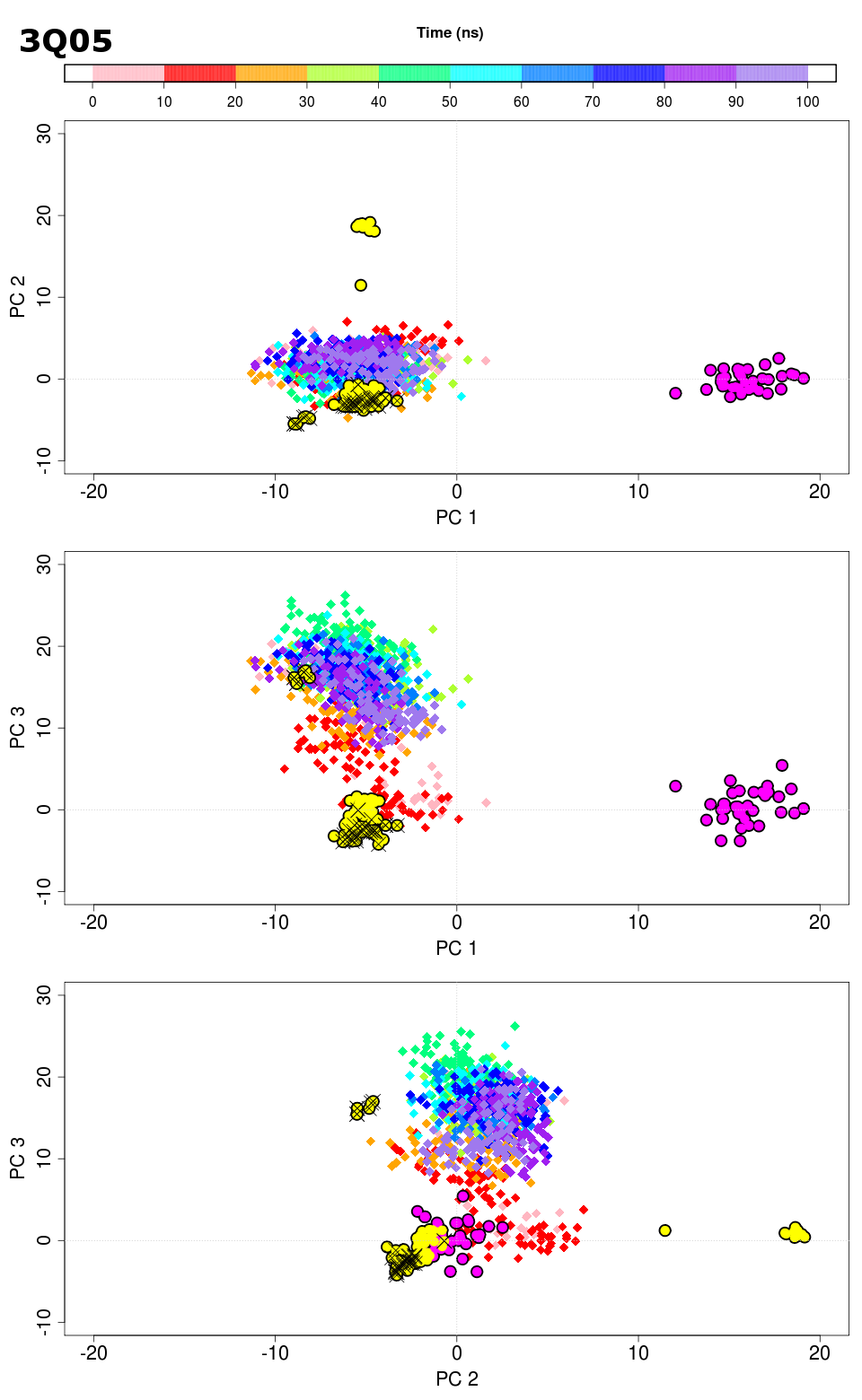

Supplement: Figures S8 — Projection of MD conformers (diamonds) onto the first three PCs defined by the experimentally-determined crystal (yellow circles) and NMR (magenta circles) structures (see Figure 2). The MD conformers are color mapped based on simulation time from 0 to 100 ns. The PDB code of starting structure used for each MD simulation is indicated on upper left. (TIFF) [file pone.0080221.s008.tiff]

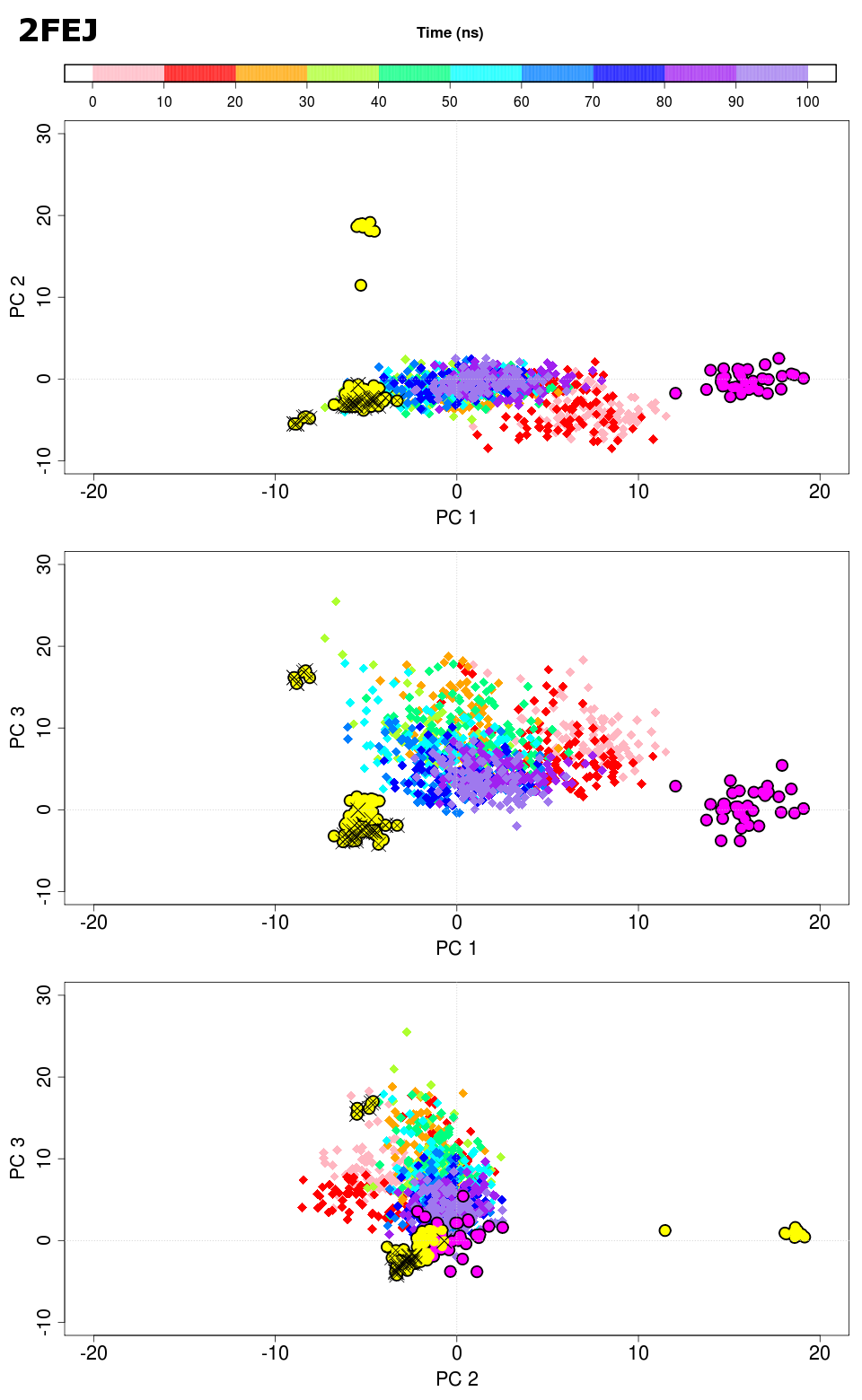

Supplement: Figures S9 — Projection of MD conformers (diamonds) onto the first three PCs defined by the experimentally-determined crystal (yellow circles) and NMR (magenta circles) structures (see Figure 2). The MD conformers are color mapped based on simulation time from 0 to 100 ns. The PDB code of starting structure used for each MD simulation is indicated on upper left. (TIFF) [file pone.0080221.s009.tiff]

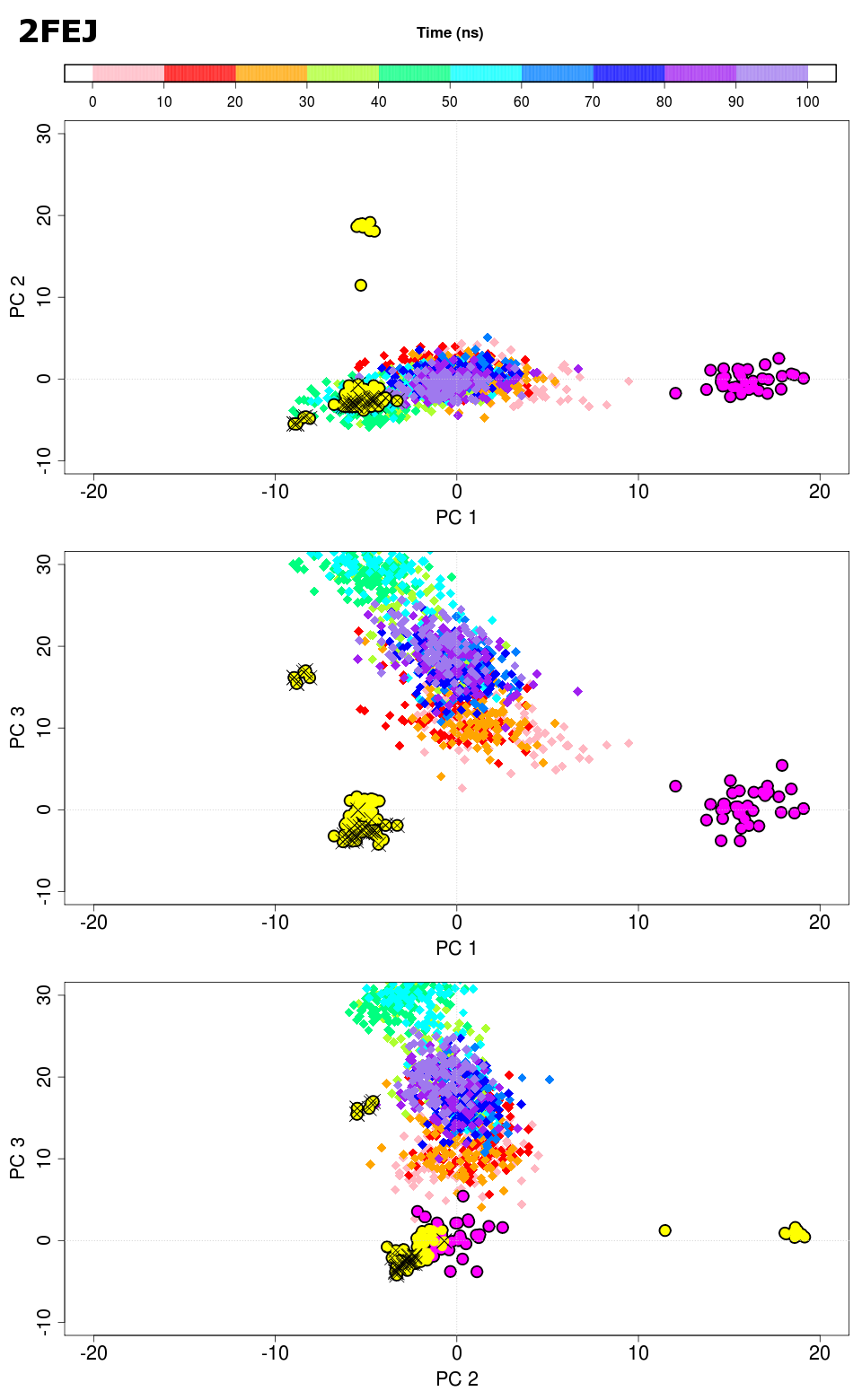

Supplement: Figures S10 — Projection of MD conformers (diamonds) onto the first three PCs defined by the experimentally-determined crystal (yellow circles) and NMR (magenta circles) structures (see Figure 2). The MD conformers are color mapped based on simulation time from 0 to 100 ns. The PDB code of starting structure used for each MD simulation is indicated on upper left. (TIFF) [file pone.0080221.s010.tiff]

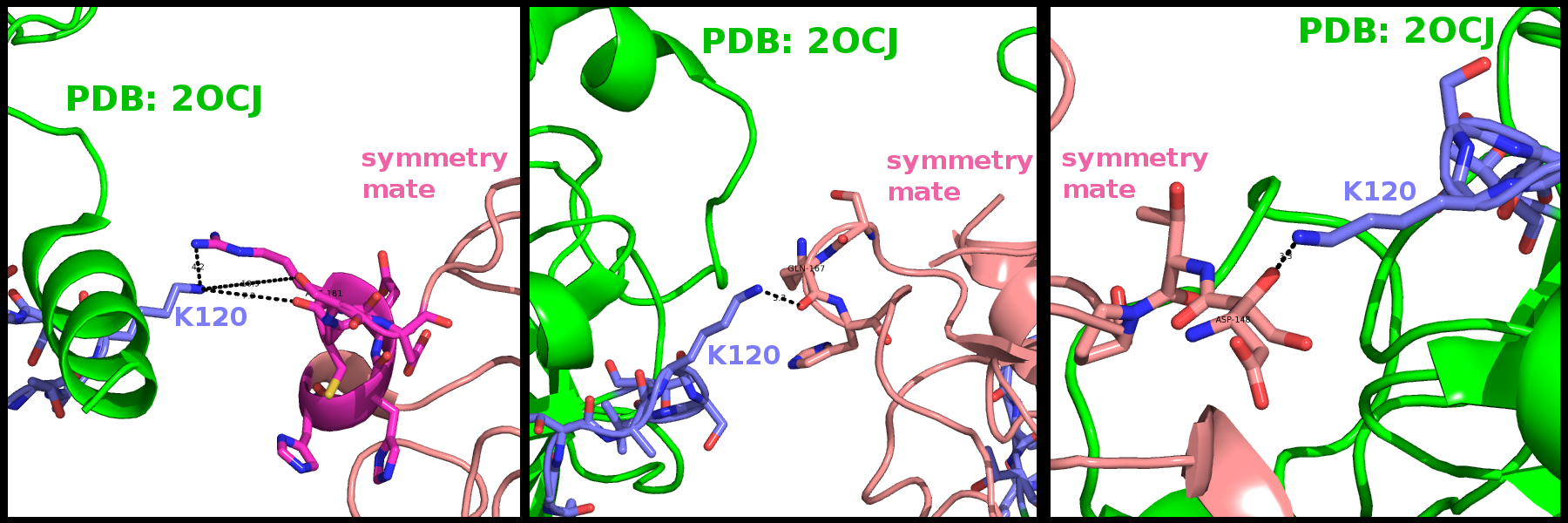

Supplement: Figure S11 — The contacts between K120 of DNA-free DBD crystal structure and residues of 3 different crystal symmetry mates (in each panel). (TIFF) [file pone.0080221.s011.tiff]

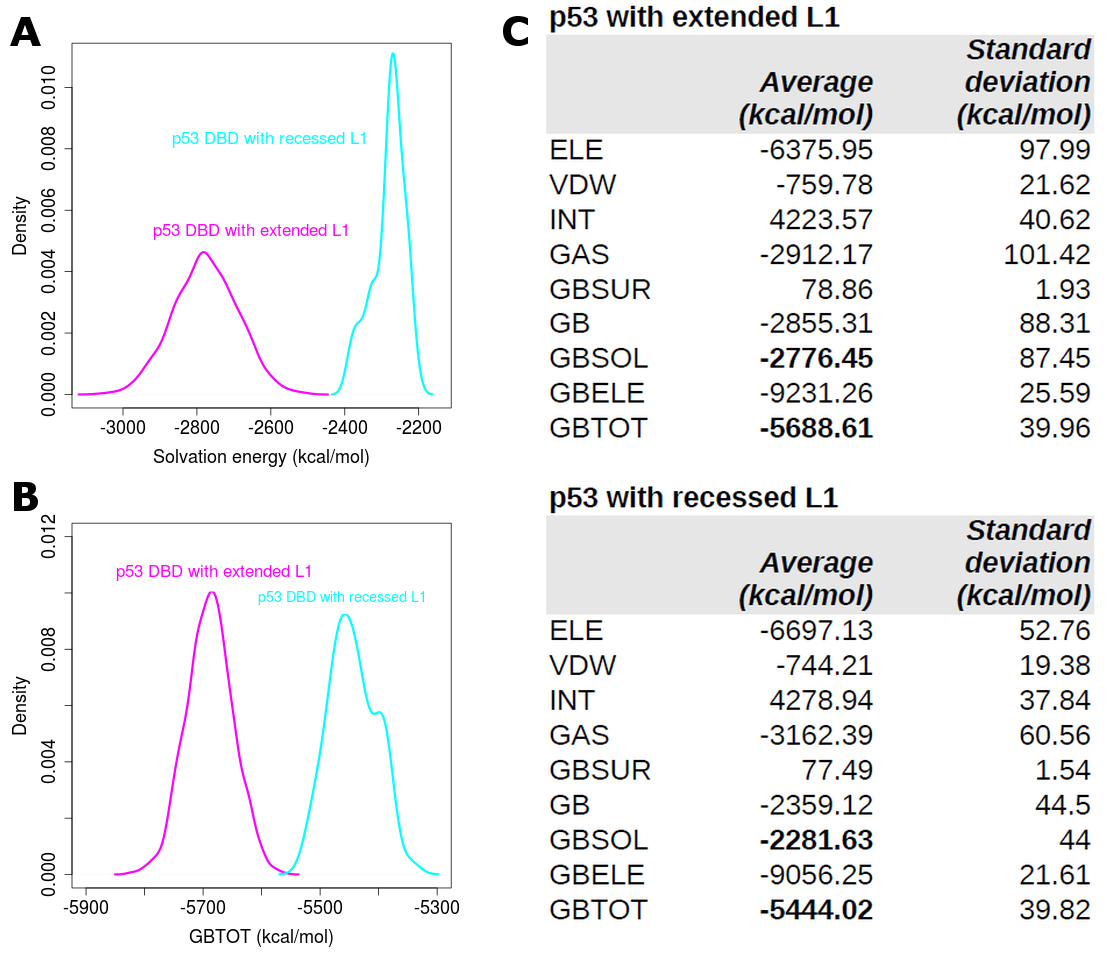

Supplement: Figure S12 — Comparison of energetic profiles of p53 DNA binding domain with extended and recessed loop 1. The energetic distribution of (A) solvation energy (B) total molecular mechanics energy of p53 DBDs with extended and recessed L1. (C) The average and standard deviations of the energetic components. ELE is the non-bonded electrostatic energy. VDW is the non-bonded van der Waals energy. INT is the sum of bond, angle and dihedral energies. GAS is the sum of ELE, VDW and INT, i.e. the molecular mechanical energy in vacuum. GBSUR is the hydrophobic contribution to solvation free energy for generalized Born (GB) calculation. (TIFF) [file pone.0080221.s012.tiff]

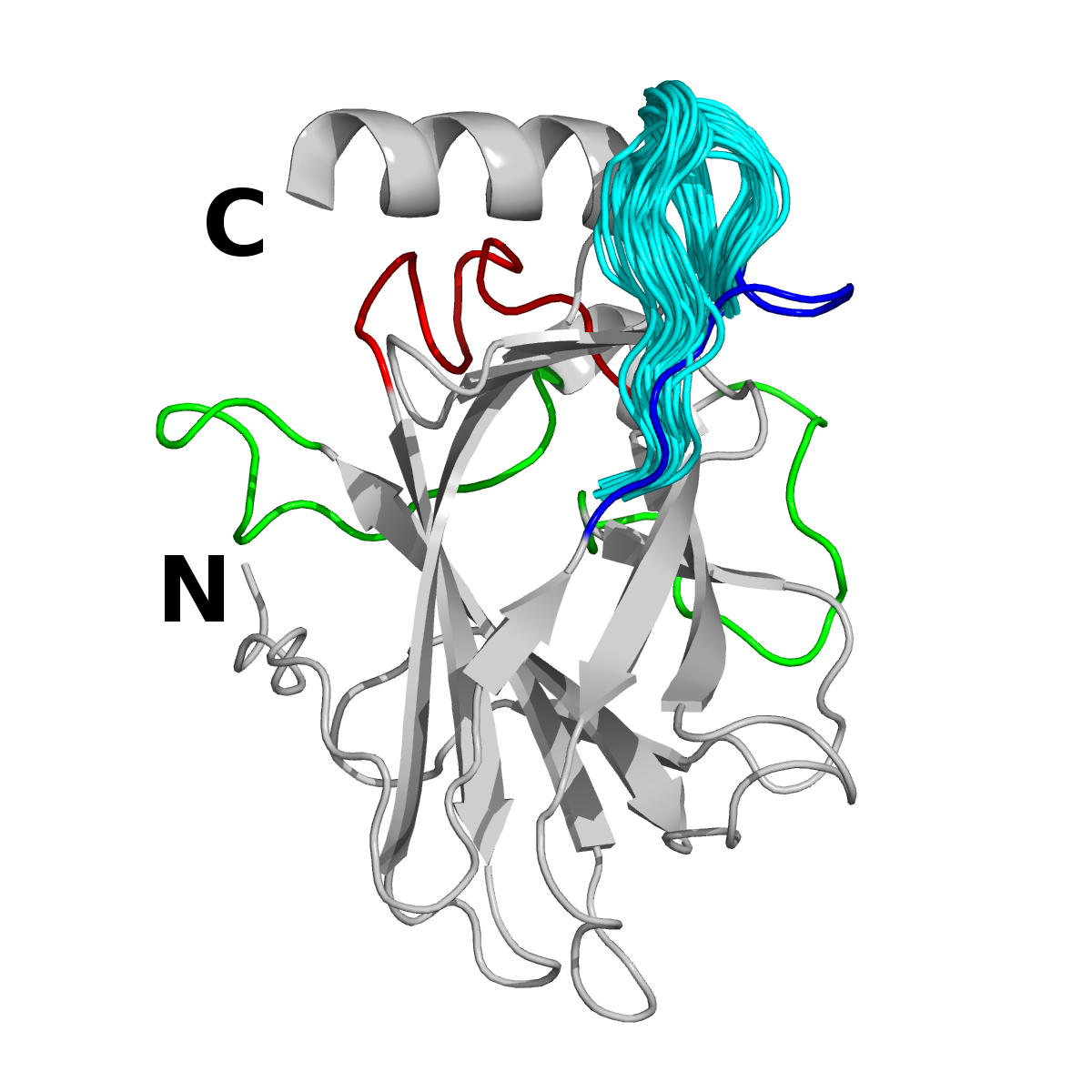

Supplement: Figure S13 — None of NMR conformers (PDB code 2FEJ) adopts recessed L1 conformation, their L1 is colored in cyan. For clarity, only L1 of NMR conformers is shown. Recessed L1 of PDB code 3Q05 chain A is colored blue. L2 and L3 are colored green and red, respectively. (TIFF) [file pone.0080221.s013.tiff]

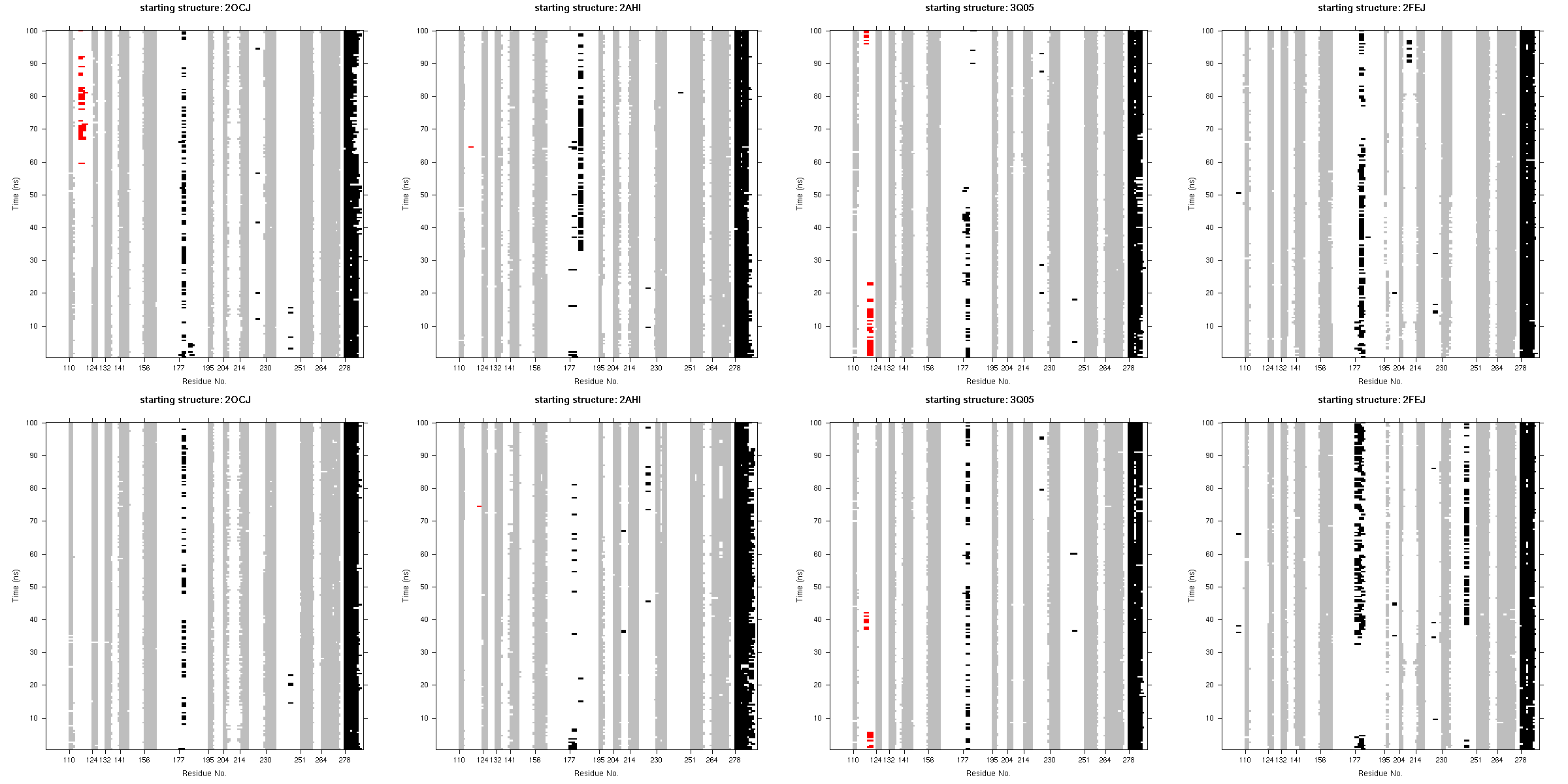

Supplement: Figure S14 — The time-dependent secondary structure profiles of MD conformers, with α helices and β sheets shown in black and grey respectively, except that the transient helix present in L1 is shown in red. The PDB code of starting structure used for each MD simulation is indicated on the top of each panel. (TIFF) [file pone.0080221.s014.tiff]

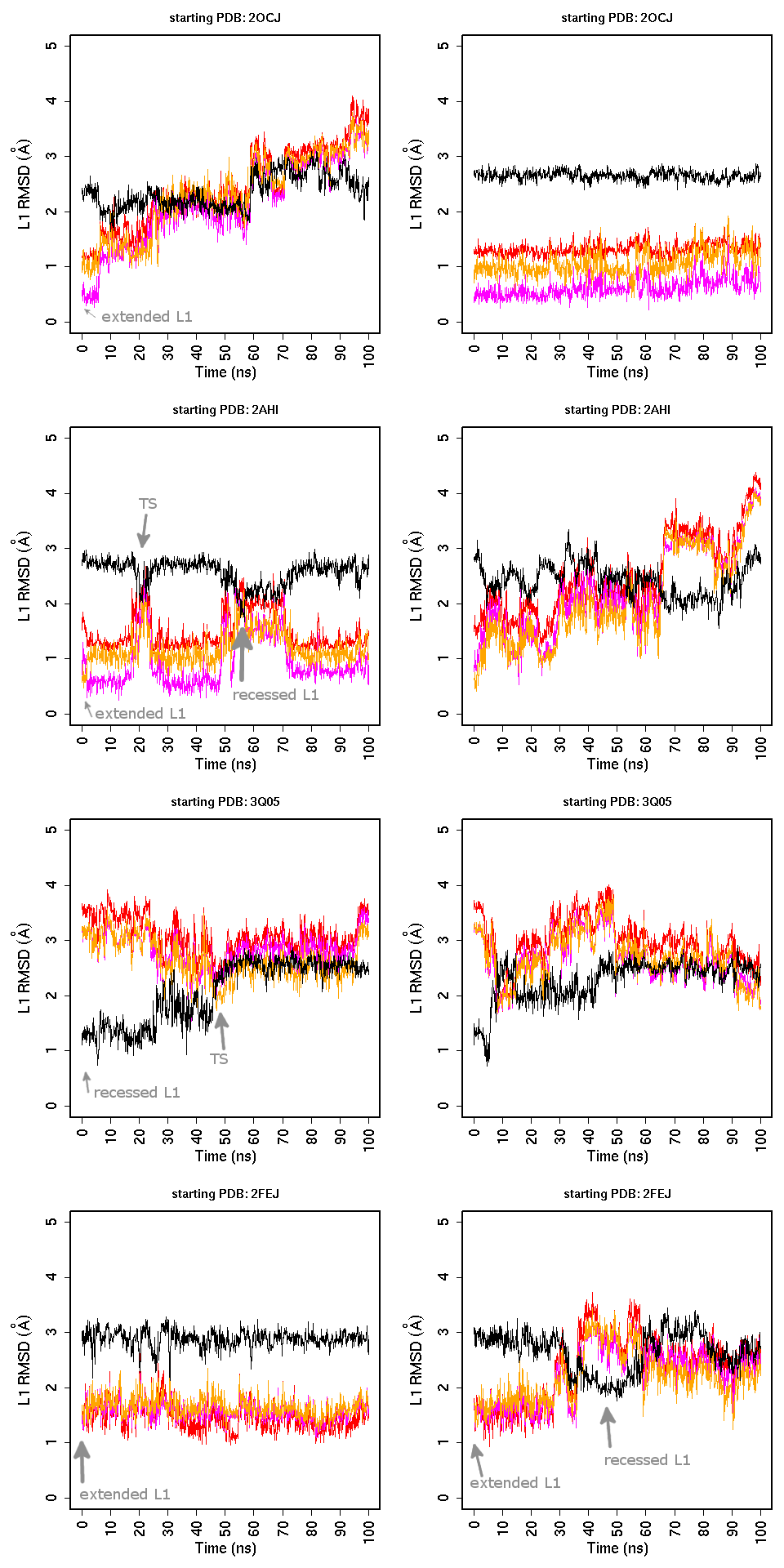

Supplement: Figure S15 — The RMSDs of L1 residues in MD conformers with respect to the L1 residues in the crystal structures with extended L1 (PDB code s 2OCJ and 2AHI in magenta and orange, respectively) and recessed L1 (PDB code 3Q05 in black). The L1 RMSDs with respect to the L1 residues in the NMR structure (PDB code 2FEJ) are plotted in red. TS: Transitioning state between extended and recessed L1. (TIFF) [file pone.0080221.s015.tiff]

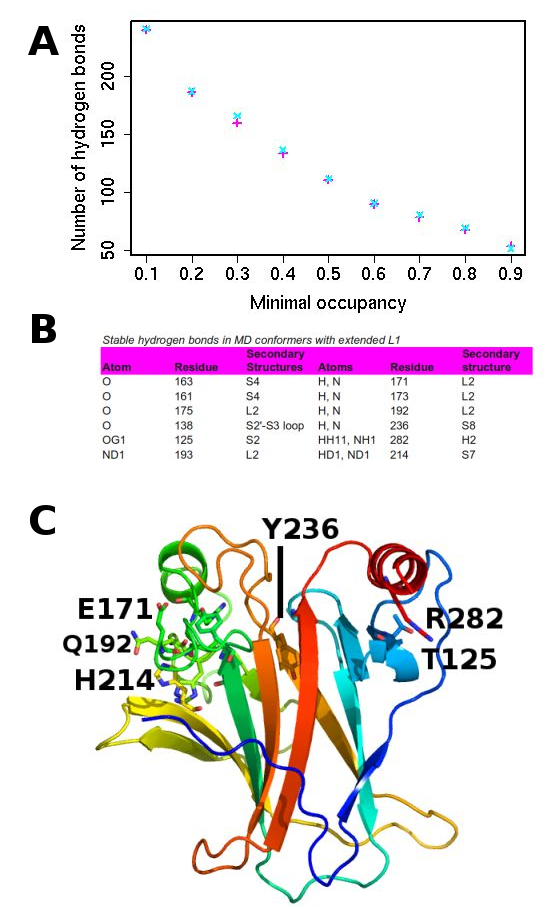

Supplement: Figure S16 — Hydrogen bonds in p53 DNA binding domain. (A) The total number of hydrogen bonds in the p53 DBD MD conformers with extended L1 (magenta +) and recessed L1 (cyan x). (B) Stable hydrogen bonds that are present in MD conformers with extended L1 but are lost in MD conformers with recessed L1. (C) The mapping of residues involved in stable hydrogen bonds onto the crystal structure with extended L1 (PDB code 2OCJ). The secondary structure coloring is the same as in Figure 1C. (TIFF) [file pone.0080221.s016.tiff]

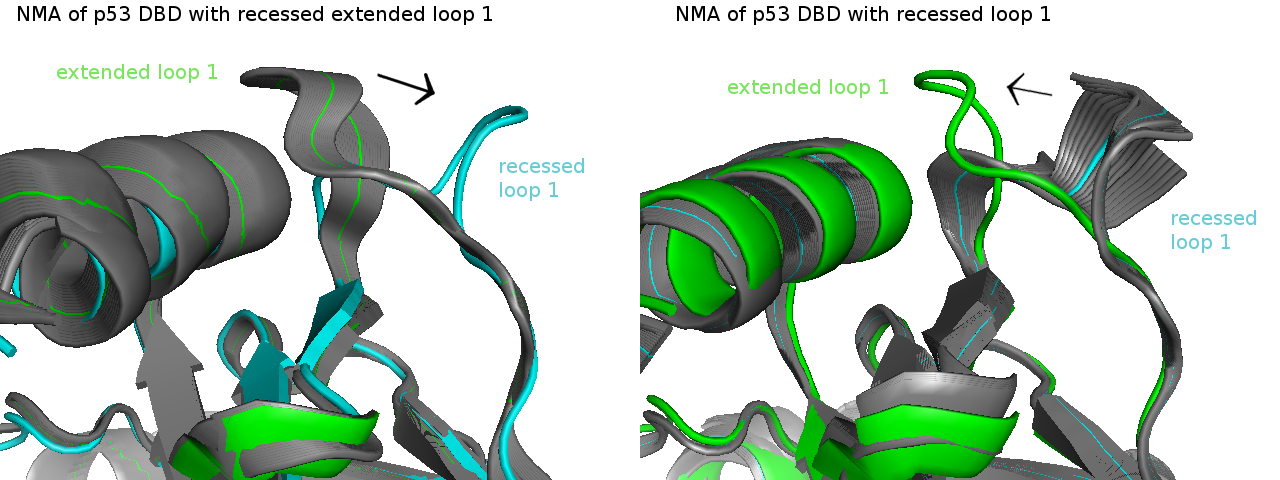

Supplement: Figure S17 — Visualization of dominant motions (gray) obtained from NMA of p53 DBD with extended (green) and recessed (cyan) loop 1. (TIF) [file pone.0080221.s017.tif]
