# Supplementary material for: Mapping the Structural and Dynamical Features of Multiple p53 DNA Binding Domains: Insights into Loop 1 Intrinsic Dynamics
Source: PLoS One. 2013 Nov 12;8(11):e80221. doi: 10.1371/journal.pone.0080221 (PMC3855832; doi:10.1371/journal.pone.0080221)
Supplement: File S1 — (DOC) [file pone.0080221.s018.doc]

**Supplementary Information: Mapping the structural and dynamical features of multiple p53 DNA binding domains: insights on loop 1 intrinsic dynamics**

Suryani Lukman, David P. Lane, Chandra S. Verma

## Sampling of extended and recessed loop 1 conformational states

To the best of our knowledge, our study is the first to distinguish the conformational states of extended and recessed loop 1 through multiple copies of MD simulations. We have used 4 different starting structures from PDB: 2OCJ, 2AHI, 3Q05 and 2FEJ. 3Q05 structure has recessed loop 1 whereas the rest of structures have extended loop 1. Interestingly, we observed the transitioning between extended and recessed loop 1 in our MD simulations regardless of the loop 1 conformation in the starting structures, this result inspired us to investigate their different dynamic behavior to further understand the possible implications in their functions.

Table S1: The conformational states of extended and recessed loop 1 that are sampled in each of 100 ns MD simulations.

| **Starting PDB structure** | **Loop 1 in starting structure** | **Trajectory** | **Extended loop 1 (%)** | **Recessed Loop 1 (%)** |
| --- | --- | --- | --- | --- |
| 20CJ | extended | 1 | 40.1 | 10.6 |
| 20CJ | extended | 2 | 100 | 0 |
| 2AHI | extended | 3 | 97 | 1.7 |
| 2AHI | extended | 4 | 44 | 7.8 |
| 3Q05 | recessed | 5 | 0.7 | 42.1 |
| 3Q05 | recessed | 6 | 5.5 | 18.9 |
| 2FEJ | extended | 7 | 98.4 | 0.4 |
| 2FEJ | extended | 8 | 32.9 | 5.6 |

## Synergistic effects of DNA and p53 conformational dynamics

To regulate cellular integrity at the transcription level, p53 DBD binds to a specific double-stranded DNA sequence of two copies of decameric half-site palindromes of the motif 5'-PuPuPuC(A/T)(T/A)GPyPyPy-3' separated by a variable spacer of 0-13 base pairs, where Purine or Pu includes A and G; Pyrimidine or Py includes C and T [1]. The C at the position 4 and the G at position 7 were previously thought to be essential yet they are replaceable in some response elements for p53 [2].

Typically, sequence-specific DNA-binding proteins undergo major conformational changes upon binding to DNA [3,4,5], but p53 DBD structures display no major conformational changes upon complexing with DNA [6,7]. On the other hand, p53-DNA complexes of p53 structures containing both DBD and TET domains display conformational dynamics in both DNA (e.g. bending) and p53 DBD (extended and recessed L1), which possibly arise from synergistic effects. Although sequence-specific DNA is a target and hence a ligand of p53, it is also possible that DNA can act as an allosteric modulator for p53 [8,9]. As of January 2012, there were 17 PDB entries containing both p53 and DNA fragments crystallized from both human and mouse species (Table S2). We analyzed these structures based on (1) the length of co-crystallized p53 sequence, (2) the co-crystallized DNA motif, (3) the length of co-crystallized DNA sequence.

Interestingly, the axial bending and twisting of DNA are more prevalent upon the binding of full-length p53 to the DNA than the binding of isolated p53 DBD [10], in which the full-length p53 induces bending in the range of 51-57° whereas p53 DBD induces bending in the range of 32-36° [10]. Although no full length p53 has been successfully co-crystallized with DNA *hitherto*, among the 17 PDB entries analyzed in this study, DNA bending is mainly observed in the complexes with longer p53 structures that contain both DBD and TET domains (PDB accession codes 3Q05, 3Q06, 3TS8) (Table S2).

Molecular dynamics simulations have also shown that the binding of DBD to DNA induces bending of DNA in the range of 20-30° and the extent of the bending depends on the DNA sequence [11]. The binding of p53 seems to prefer DNA sequences containing CATG at the center of both half sites over DNA sequences containing at least a CAAG or CTTG at the center of the half sites [12]. Consequently, the higher affinity of p53-DNA results in greater bending of the DNA [11]. Among the 17 PDB entries analyzed in this study, only the PDB accession code 1TSR has a CTTG and a CAAG at the center of the half sites, whereas others have CATG at the center of the half sites. Longer DNA (e.g. PDB accession code 3KZ8, 3Q05, and 3Q06) also tends to contain two copies of CATG, which possibly contribute to stronger p53-DNA binding and hence the observed DNA bending (in PDB accession code 3Q05 and 3Q06). The DNA bending in turn allows the adoption of recessed L1 conformation in p53 DBD in order to avoid steric clash with the DNA [13]. Moreover, a study using fluorescence anisotropy and analytical ultracentrifugation showed that at least a 20 bp specific DNA sequence must interact with p53 in order to form a stable p53-DNA complex [14], highlighting the importance of the length of the specific DNA sequence. The length of the spacer separating the two decamers also matters, where DNA with no spacer base pair binds p53 stronger than the DNA with spacer base pairs [15] and more than half of the high-probability binding sites of p53 DNA targets have no spacer base pairs [16].

Given that the p53 affinities for specific and non-specific DNA differ by less than 10-fold [14,17], recent studies [13,17] proposed that the differences in binding off rates (due to conformational changes) contribute to the duration of p53-DNA binding. It is possible that L1 of p53 DBD can adopt distinct conformations depending on the particular DNA sequence, which may be reflected in the different off rates of p53-DNA complexes. Indeed, indirect support is provided by our MD simulations of DNA-free p53 which can sample a much larger conformational space of L1 than those seen in the experimentally determined structures. Future studies employing MD simulations for both p53 and distinct sequence-specific (target) DNA will provide more insights on the synergistic and allosteric aspects of p53-DNA conformational dynamics and changes.

# REFERENCES

1. el-Deiry WS, Kern SE, Pietenpol JA, Kinzler KW, Vogelstein B (1992) Definition of a consensus binding site for p53. Nat Genet 1: 45-49.

2. Menendez D, Inga A, Resnick MA (2009) The expanding universe of p53 targets. Nat Rev Cancer 9: 724-737.

3. Frankel AD, Kim PS (1991) Modular structure of transcription factors: implications for gene regulation. Cell 65: 717-719.

4. Alber T (1993) Protein-DNA interactions: how GCN4 binds DNA. Curr Biol 3: 182-184.

5. Spolar RS, Record MT, Jr. (1994) Coupling of local folding to site-specific binding of proteins to DNA. Science 263: 777-784.

6. Cho Y, Gorina S, Jeffrey PD, Pavletich NP (1994) Crystal structure of a p53 tumor suppressor-DNA complex: understanding tumorigenic mutations. Science 265: 346-355.

7. Kitayner M, Rozenberg H, Kessler N, Rabinovich D, Shaulov L, et al. (2006) Structural basis of DNA recognition by p53 tetramers. Mol Cell 22: 741-753.

8. Lefstin JA, Yamamoto KR (1998) Allosteric effects of DNA on transcriptional regulators. Nature 392: 885-888.

9. Phillips K, Luisi B (2000) The virtuoso of versatility: POU proteins that flex to fit. J Mol Biol 302: 1023-1039.

10. Nagaich AK, Zhurkin VB, Durell SR, Jernigan RL, Appella E, et al. (1999) p53-induced DNA bending and twisting: p53 tetramer binds on the outer side of a DNA loop and increases DNA twisting. Proc Natl Acad Sci U S A 96: 1875-1880.

11. Pan Y, Nussinov R (2007) Structural basis for p53 binding-induced DNA bending. J Biol Chem 282: 691-699.

12. Weinberg RL, Veprintsev DB, Bycroft M, Fersht AR (2005) Comparative binding of p53 to its promoter and DNA recognition elements. J Mol Biol 348: 589-596.

13. Emamzadah S, Tropia L, Halazonetis TD (2011) Crystal structure of a multidomain human p53 tetramer bound to the natural CDKN1A (p21) p53-response element. Mol Cancer Res 9: 1493-1499.

14. Weinberg RL, Veprintsev DB, Fersht AR (2004) Cooperative binding of tetrameric p53 to DNA. J Mol Biol 341: 1145-1159.

15. Kitayner M, Rozenberg H, Rohs R, Suad O, Rabinovich D, et al. (2010) Diversity in DNA recognition by p53 revealed by crystal structures with Hoogsteen base pairs. Nat Struct Mol Biol 17: 423-429.

16. Wei CL, Wu Q, Vega VB, Chiu KP, Ng P, et al. (2006) A global map of p53 transcription-factor binding sites in the human genome. Cell 124: 207-219.

17. Petty TJ, Emamzadah S, Costantino L, Petkova I, Stavridi ES, et al. (2011) An induced fit mechanism regulates p53 DNA binding kinetics to confer sequence specificity. EMBO J 30: 2167-2176.
